# Sex difference in survival benefits of immune checkpoint inhibitors in advanced non-small cell lung cancer: A meta-analysis

**DOI:** 10.1371/journal.pone.0361953

**Published:** 2026-11-03

**Authors:** Tingting Liu, Dandan Song, Jun Chen, Jiacheng Yao, Jiehao Liao, Jun Dang

**Affiliations:** 1 Department of Radiation Oncology, The First Hospital of China Medical University, Shenyang, China; 2 Department of Radiation Oncology, Anshan Cancer Hospital, Anshan, China; 3 Department of Radiation Oncology, Xiang’an Hospital of Xiamen University, School of Medicine, Xiamen University, Xiamen, China; 4 Department of Radiation Oncology, Shenyang Tenth People’s Hospital, Shenyang, China; 5 Department of Oncology, The Seventh Affiliated Hospital, Sun Yat-sen University, Shenzhen, China; Hokkaido University: Hokkaido Daigaku, JAPAN

## Abstract

**Background:**

Whether sex influences the survival benefit of immune checkpoint inhibitors (ICIs) in advanced non-small cell lung cancer (NSCLC) remains uncertain.

**Methods:**

We systematically searched PubMed, Embase, Cochrane Library, Web of Science, and Scopus up to April 1, 2026, for randomized controlled trials (RCTs) comparing ICIs with chemotherapy and reporting overall survival (OS) or progression-free survival (PFS) stratified by sex. A random-effects model was used for meta-analysis.

**Results:**

Sixty-three publications (reporting 42 phase 3 RCTs) involving 25,827 patients (18,772 male; 7,055 female) were included. Compared with chemotherapy, ICIs significantly improved both OS and PFS in males (OS: HR 0.74 [95% CI 0.71–0.78]; PFS: HR 0.59 [95% CI 0.54–0.64]) and females (OS: HR 0.83 [95% CI 0.77–0.90]; PFS: HR 0.76 [95% CI 0.66–0.86]). Pooled treatment-by-sex interaction analyses demonstrated significantly greater OS (interaction HR = 0.90 [95% CI 0.84–0.97]; P = 0.01) and PFS (interaction HR = 0.82 [95% CI 0.73–0.92]; P = 0.0006) benefits in males than in females. In subgroup analyses, sex‑based differences in OS reached statistical significance in patients receiving ICI monotherapy (P = 0.02), including those treated with anti‑PD‑1 monotherapy (P = 0.04) and those with PD‑L1 expression ≥50% (P = 0.02). However, these differences did not persist after the False Discovery Rate (FDR) correction (adjusted P = 0.07, 0.10, and 0.07, respectively). In contrast, sex‑based differences in PFS remained statistically significant after FDR correction in the ICI monotherapy (adjusted P = 0.001), anti‑PD‑1 monotherapy (adjusted P = 0.001), and PD‑L1 ≥ 50% (adjusted P = 0.004) subgroups. No significant sex‑based differences in either OS or PFS were observed among patients treated with ICI plus chemotherapy.

**Conclusions:**

Although ICIs are superior to chemotherapy in both sexes, the PFS benefit of ICI monotherapy appears to be attenuated in females relative to males. Given the imbalances in baseline confounders coupled with the underrepresentation of females in the included trials, these findings should be interpreted with caution and warrant confirmation in future RCTs designed specifically to investigate sex‑based differences.

## Introduction

Currently, immune checkpoint inhibitors (ICIs) represent the preferred treatment for advanced non-small cell lung cancer (NSCLC) lacking targetable oncogenic driver mutations. Among potential biomarkers for ICI response, programmed cell death ligand-1 (PD-L1) expression on tumor cells has been clinically validated as a predictor of treatment efficacy. Additionally, patient characteristics such as age [1] and smoking status [2] have also been associated with ICI efficacy.

Sex represents another biological factor potentially influencing ICI outcomes. Preclinical studies indicate that sex hormones can regulate the PD-1/PD-L1 axis and modulate PD-L1 expression [3]. Furthermore, comprehensive analyses of genome-wide transcriptome data and tumor samples in NSCLC have revealed sex-specific differences in antitumor immunity and immune evasion mechanisms [4]. Nevertheless, the clinical relevance of sex difference in ICI efficacy remains controversial. Although multiple meta-analyses [5–19] have investigated this association across various malignancies, their findings have been inconsistent. These discrepancies may arise from methodological limitations, including restricted sample sizes, inadequate follow-up durations, and the inclusion of heterogeneous cancer types, all of which could compromise the reliability of pooled estimates.

Given the recent publication of numerous randomized controlled trials (RCTs) and updated long-term survival data, a contemporary synthesis of the evidence is warranted. Therefore, we conducted an updated systematic review and meta-analysis to clarify the impact of sex on ICI treatment outcomes in advanced NSCLC.

## Methods

### Literature search and inclusion criteria

We conducted a systematic review and meta-analysis in accordance with the Preferred Reporting Items for Systematic Reviews and Meta-Analyses (PRISMA) 2020 statement [20]. Two authors (TL and DS) independently searched PubMed, Embase, the Cochrane Library, Web of Science, and Scopus for eligible studies published up to April 1, 2026. The search strategy and specific terms are detailed in S1 Table (S3 File). Studies were included based on the following criteria: (1) phase 2 or 3 RCTs comparing ICIs (administered as monotherapy or in combination) with chemotherapy in patients with advanced NSCLC; and (2) availability of overall survival (OS) or progression-free survival (PFS) data stratified by sex. When multiple publications originated from the same RCT, the publication reporting the most recent or comprehensive data was selected.

### Data extraction and risk of bias assessment

Two authors (TL and DS) independently extracted the following data: (1) trial characteristics: trial name, first author, publication year, study design, geographical region, number of patients by sex, median follow-up duration, ICI agent, CT regimen, treatment modality (monotherapy/combination), and treatment line; (2) patient characteristics: age, Eastern Cooperative Oncology Group (ECOG) performance status, histological subtype, and PD-L1 expression level; (3) outcomes: hazard ratios (HRs) with corresponding 95% confidence intervals (CIs) for OS and PFS, stratified by sex.

The risk of bias of the included trials was assessed using the Cochrane Risk of Bias Tool [21], which consists of the following domains: sequence generation, allocation concealment, blinding, incomplete data, selective reporting, and other bias. The trials were finally classified as low (all domains indicated as low risk), high (one or more domains indicated as high risk), and unclear risk of bias (more than three domains indicated as unclear risk).

### Statistical analysis

The primary outcome was OS. Statistical analyses were performed using R software (version 4.5.3) with the meta package. Given the anticipated clinical and methodological heterogeneity across the included studies, pooled HRs were calculated using a random-effects model with inverse-variance weighting. The between-study variance (τ^2^) was estimated using the restricted maximum likelihood (REML) estimator in the primary meta-analyses.

For meta-analyses involving a limited number of studies (n ≤ 5), we applied the Hartung-Knapp-Sidik-Jonkman (HKSJ) method [22,23] to adjust the confidence intervals. This method employs a variance correction and a t-distribution with k − 1 degrees of freedom, yielding more conservative interval estimates when between-study heterogeneity is imprecisely estimated. For trials with two intervention arms sharing a common control group (IMpower-150, POSEIDON, and RATIONALE-307), a multivariate meta-analysis approach [24,25] was used to pool the two HRs; this approach accounts for the correlation between comparisons and avoids treating the shared control arm as independent information.

Heterogeneity was assessed using Cochran’s Q test and quantified by the I^2^ statistic, with values of 25%, 50%, and 75% considered to indicate low, moderate, and high heterogeneity, respectively. Meta-regression analysis was conducted to explore potential sources of heterogeneity. Pre-specified subgroup analyses were performed when at least three trials were available, stratified by race, histology, PD-L1 expression level, treatment modality (ICI monotherapy vs. combination therapy), and treatment line (first-line vs. subsequent lines).

To quantify the differential efficacy of ICIs between sexes, we first calculated the treatment-by-sex interaction HR for each trial as the ratio of the male-specific HR (ICI vs. chemotherapy) to the female-specific HR; these trial-specific ratios were then pooled using a random-effects model, with a two-sided P < 0.05 considered statistically significant. To account for multiple testing in subgroup interaction analyses, we applied the False Discovery Rate (FDR) correction [26,27] to adjust P-values.

Sensitivity analyses were performed to evaluate the robustness of the primary findings, including leave-one-out analyses (sequentially omitting one trial at a time) and analyses excluding trials with a critically low number of female patients (n ≤ 30). Publication bias was assessed using funnel plots and Egger’s regression test [28].

## Results

### Study selection and characteristics

The initial literature search yielded 6582 records. After screening titles and abstracts, 6483 articles were excluded. The remaining 99 full-text articles were assessed for eligibility. Ultimately, 63 publications [29–91], reporting data from 42 phase 3 RCTs involving a total of 25,827 patients (18,772 male; 7,055 female), were included in the meta-analysis. The study selection process is summarized in Fig 1. Among the 42 phase 3 RCTs included, 15 were conducted in Asian countries, 6 in non-Asian countries, and 21 involved multi-regional populations. Twenty RCTs evaluated ICI monotherapy and twenty-two evaluated ICI combination therapy. ICIs were administered as first-line (1L) therapy in 32 RCTs and as second- or later line (≥2L) therapy in 10 RCTs. Common chemotherapy regimens were cisplatin or carboplatin combined with pemetrexed or paclitaxel in the 1L setting, and docetaxel in the ≥ 2L setting. Most patients had an ECOG score of 0–1. The proportion of never smokers was 17% (n = 4343). The median sample size per trial was 380 (interquartile range [IQR], 318–532) for males and 116 (IQR, 88–217) for females. The median follow-up duration across trials was 26.0 months (IQR, 16.3–34.4). Key trial characteristics and survival outcomes are summarized in Table 1. We have deposited the raw data extraction matrix (an Excel file containing individual trial‑level hazard ratios, standard errors, and sample sizes) in the Zenodo open repository (https://doi.org/10.5281/zenodo.21437774).

**Table 1:** Characteristics and outcomes of included trials.

Trial name/year	Region	Primary endpoint	Median follow-up (month)	Age (ICI/CT) (median)	Histological type	PD-L1 level	Treatment line	Sex	Treatment (sample size)	HR(95%CI) for OS	HR(95%CI) for PFS
CheckMate-017/2015 [29]	Muti	OS	11.1	62/64	SCC	NA	≥2	Male	Niv/CT(111/97)	0.57(0.41-0.78)	0.63(0.46-0.85)
								Female	Niv/CT(24/40)	0.67(0.36-1.25)	0.71(0.40-1.26)
CheckMate-057/2015 [30]	Muti	OS	13.2	61/64	non-SCC	NA	≥2	Male	Niv/CT(151/168)	0.73(0.56-0.96)	0.81(0.63-1.04)
								Female	Niv/CT(141/122)	0.78(0.58-1.04)	1.04(0.80-1.37)
CheckMate-026/2017 [31]	Muti	PFS	13.5	63/65	NSCLC	≥1%	1	Male	Niv/CT(184/148)	0.97(0.74-1.26)	1.05(0.81-1.37)
								Female	Niv/CT(87/122)	1.15(0.79-1.66)	1.36(0.98-1.90)
CheckMate-078/2015 [32], 2021 [33]	Muti	OS	25.9	60/60	NSCLC	NA	≥2	Male	Niv/CT(263/134)	0.75(0.61-0.92)	0.72(0.59-0.89)
								Female	Niv/CT(75/32)	0.79(0.53-1.18)	1.08(0.71-1.65)
CheckMate-227/2019 [34]	Multi	OS	29.3	64/64	NSCLC	NA	1	Male	Niv + Ipi/CT(393/385)	0.68(0.57-0.80)	NR
								Female	Niv + Ipi/CT(190/198)	0.89(0.70-1.12)	NR
CheckMate-227 (part2)/2023 [35]	Multi	OS	19.5	64/64	NSCLC	NA	1	Male	Niv + CT/CT(264/266)	0.80(0.65-0.99)	NR
								Female	Niv + CT/CT(113/112)	0.84(0.60-1.19)	NR
CheckMate-9LA/ 2021 [36], 2025 [37]	Multi	OS	64.5	65/65	NSCLC	NA	1	Male	Niv + Ipi + CT/CT(252/252)	0.73(0.60-0.88)	0.67(0.55-0.81)
								Female	Niv + Ipi + CT/CT(109/106)	0.79(0.59-1.06)	0.81(0.60-1.10)
TASUKI-52/ 2021 [38], 2025 [39]	Multi	PFS	36.1	66/66	non-SCC	NA	1	Male	Niv + Bev + CT/Bev + CT(205/206)	NR	0.55 (0.43-0.71)
								Female	Niv + Bev + CT/Bev + CT(70/69)	NR	0.78 (0.50-1.22)
Keynote-010/2016 [40], 2021 [41]	Muti	OS	67.4	63/63	NSCLC	≥1%	≥2	Male	Pem/CT (425/209)	0.71(0.60-0.86)	0.78(0.64-0.94)
								Female	Pem/CT (265/134)	0.66(0.53-0.84)	1.02(0.78-1.32)
Keynote-024/2016 [42], 2019 [43]	Muti	PFS	25.2	65/66	NSCLC	≥50%	1	Male	Pem/CT(92/95)	0.54(0.36-0.79)	0.39(0.26-0.58)
								Female	Pem/CT(62/56)	0.95(0.56-1.62)	0.75(0.46-1.21)
Keynote-042/ 2019 [44]	Muti	OS	12.8	63/63	NSCLC	≥1%	1	Male	Pem/CT(450/452)	0.80(0.68-0.94)	NR
								Female	Pem/CT(187/185)	0.89(0.68-1.17)	NR
Keynote-189/2018 [45]	Multi	OS/PFS	10.5	65/64	non-SCC	NA	1	Male	Pem + CT/CT (254/109)	0.70(0.50-0.99)	0.66(0.50-0.87)
								Female	Pem + CT/CT (156/97)	0.29(0.19-0.44)	0.40(0.29-0.54)
Keynote-407/2018 [46]	Multi	OS/PFS	7.8	65/65	SCC	NA	1	Male	Pem + CT/CT (220/235)	0.69(0.51-0.94)	0.58(0.46-0.73)
								Female	Pem + CT/CT (58/46)	0.42(0.22-0.81)	0.49(0.30-0.81)
Keynote-033/2023 [47]	Multi	OS/PFS	22.3	61/61	NSCLC	≥1%	≥2	Male	Pem/CT (157/164)	0.71(0.55-0.93)	NR
								Female	Pem/CT (56/48)	0.90(0.55-1.47)	NR
IMpower-110/ 2020 [48], 2021 [49]	Multi	OS	31.3	63/66	NSCLC	≥50%	1	Male	Ate/CT(79/64)	0.73(0.48-1.11)	NR
								Female	Ate/CT(28/34)	0.84(0.45-1.58)	NR
IMpower-130/2019 [50]	Multi	OS/PFS	18.5	64/65	non-SCC	NA	1	Male	Ate + CT/CT(266/134)	0.87(0.66-1.15)	0.67(0.54-0.85)
								Female	Ate + CT/CT(185/94)	0.66(0.46-0.93)	0.59(0.45-0.78)
IMpower-131/2020 [51]	Multi	OS/PFS	26.8	65/65	SCC	NA	1	Male	Ate + CT/CT(280/277)	0.91(0.75-1.12)	0.71(0.59-0.85)
								Female	Ate + CT/CT(63/63)	0.68(0.44-1.04)	0.66(0.45-0.97)
IMpower-132/2021 [52]	Multi	OS/PFS	28.4	64/63	non-SCC	NA	1	Male	Ate + CT/CT(192/192)	0.84(0.63-1.13)	0.64(0.51-0.79)
								Female	Ate + CT/CT(100/94)	0.93(0.73-1.18)	0.51(0.36-0.71)
IMpower-150/2018 [53], 2021 [54]	Multi	OS/PFS	15.5	63/63	non-SCC	NA	1	Male	Ate + Bev + CT/Bev + CT(240/239)	0.72(0.58-0.90)	0.55(0.44-0.69)
								Female	Ate + Bev + CT/Bev + CT(160/161)	0.92(0.70-1.22)	0.73(0.54-0.98)
								Male	Ate + CT/Bev + CT(241/239)	0.79(0.64-0.98)	NR
								Female	Ate + CT/Bev + CT(161/161)	0.84(0.63-1.12)	NR
IMpower-210/ 2024 [55]	China	OS	30.2	NR	NSCLC	NA	2	Male	Ate/CT(239/116)	0.77(0.59-0.99)	NR
								Female	Ate/CT(73/39)	1.06(0.67-1.67)	NR
IPSOS/2023 [56]	Multi	OS	41.0	75/75	NSCLC	NA	1	Male	Ate/CT(220/108)	0.76(0.59-0.98)	NR
								Female	Ate/CT(82/43)	0.86(0.58-1.27)	NR
OAK/2018 [57]	Muti	OS	26.0	63/64	NSCLC	NA	≥2	Male	Ate/CT(379/379)	0.79(0.66-0.93)	NR
								Female	Ate/CT(234/233)	0.81(0.65-1.01)	NR
BFAST/2022 [58]	Muti	PFS	18.2	65/66	NSCLC	≥1%	1	Male	Ate/CT(106/108)	0.97 (0.68-1.38)	0.72 (0.53-0.97)
								Female	Ate/CT(39/38)	0.68 (0.37-1.22)	0.76 (0.46-1.26)
EMPOWER-Lung 1/2021 [59], 2025 [60]	Multi	OS/PFS	35.0	63/64	NSCLC	≥50%	1	Male	Cem/CT(248/231)	0.52(0.42-0.65)	0.45(0.36-0.56)
								Female	Cem/CT(35/49)	0.92(0.52-1.62)	0.82(0.48-1.40)
EMPOWER-Lung 3/2022 [61], 2023 [62]	Multi	OS	28.4	63/63	NSCLC	NA	1	Male	Cem + CT/CT(268/123)	0.55(0.42-0.71)	0.48(0.38-0.62)
								Female	Cem + CT/CT(44/31)	0.98(0.54-1.78)	0.71(0.42-1.20)
JAVELIN Lung 200/2018 [63], 2021 [64]	Multi	OS	35.4	64/63	NSCLC	≥1%	≥2	Male	Ave/CT(182/185)	0.82(0.65-1.03)	0.77(0.59-1.01)
								Female	Ave/CT(82/80)	1.02(0.72-1.45)	1.931(1.24-3.01)
JAVELIN Lung 100/2024 [65]	Multi	OS/PFS	48.8	64/63	NSCLC	≥1%	1	Male	Ave/CT(112/158)	0.77(0.58-1.03)	0.66(0.48-0.90)
								Female	Ave/CT(39/58)	1.10(0.69-1.75)	0.94(0.56-1.58)
MYSTIC/2020 [66]	Muti	OS/PFS	30.2	64/65	NSCLC	≥25%	1	Male	Dur/CT(113/106)	0.81(0.59-1.11)	NR
								Female	Dur/CT(50/56)	0.66(0.41-1.04)	NR
POSEIDON/ 2023 [67], 2025 [68]	Multi	OS/PFS	63.4	65/64	NSCLC	NA	1	Male	Dur + CT/CT(253/248)	0.79(0.66-0.96)	0.76(0.62-0.93)
								Female	Dur + CT/CT(85/89)	0.90(0.65-1.25)	0.75(0.52-1.06)
								Male	Dur + Tre + CT/CT(269/248)	0.68(0.57-0.82)	0.67(0.54-0.82)
								Female	Dur + Tre + CT/CT(69/89)	0.92(0.65-1.31)	0.77(0.521-1.3)
NEPTUNE/2023 [69]	Multi	OS	32.9	63/65	NSCLC	NA	1	Male	Dur + Tre/CT(297/305)	0.98(0.82-1.17)	NR
								Female	Dur + Tre/CT(113/108)	1.13(0.83-1.53)	NR
PEARL/2024 [70]	Multi	OS	53.7	61/64	NSCLC	≥25%	1	Male	Dur/CT(268/269)	0.83 (0.69-1.00)	NR
								Female	Dur/CT(67/65)	0.91 (0.63-1.33)	NR
RATIONALE-303/2023 [71]	Multi	OS	16.0	61/61	NSCLC	NA	≥2	Male	Tis/CT(416/206)	0.61(0.50-0.74)	NR
								Female	Tis/CT(119/64)	0.95(0.65-1.38)	NR
RATIONALE-304/2021 [72], 2024 [73], 2025 [74]	China	PFS	16.1	60/61	non-SCC	NA	1	Male	Tis + CT/CT(168/79)	0.84(0.57-1.25)	0.53(0.37-0.75)
								Female	Tis + CT/CT(55/32)	1.02(0.48-2.18)	0.85(0.49-1.48)
RATIONALE-307/2021 [75]/2024 [76], 2026 [77]	China	PFS	44.8	60/62	SCC	NA	1	Male	Tis + CT-A/CT(107/111)	0.77(0.56-1.05)	0.49(0.36-0.69)
								Female	Tis + CT-A/CT(13/10)	0.50(0.16-1.54)	0.34(0.12-0.99)
								Male	Tis + CT-B/CT(112/111)	0.83(0.61-1.13)	0.48(0.33-0.64)
								Female	Tis + CT-B/CT(7/10)	1.10(0.35-3.42)	0.46(0.13-1.61)
CameL/2021 [78]/ 2024 [79]	China	PFS	65.2	59/61	non-SCC	NA	1	Male	Cam + CT/CT(146/149)	0.81(0.62-1.05)	0.52(0.40-0.67)
								Female	Cam + CT/CT(59/58)	0.56(0.35-0.90)	0.61(0.40-0.94)
CameL-sq/2022 [80]	China	PFS	13.5	64/62	SCC	NA	1	Male	Cam + CT/CT(179/180)	0.55(0.40-0.75)	0.35(0.27-0.46)
								Female	Cam + CT/CT(14/16)	1.06(0.21-5.26)	0.97(0.40-2.34)
GEMSTONE-302/2022 [81]	China	PFS	17.8	62/64	NSCLC	NA	1	Male	Sug + CT/CT(254/129)	NR	0.48(0.38-0.62)
								Female	Sug + CT/CT(66/30)	NR	0.60(0.37-0.99)
CHOICE-01/2023 [82], 2024 [83]	China	PFS	21.2	63/61	NSCLC	NA	1	Male	Tor + CT/CT(247/130)	0.75(0.58-0.98)	0.50(0.39-0.64)
								Female	Tor + CT/CT(62/26)	0.69(0.38-1.30)	0.58(0.35-1.00)
ASTRUM-004/2024 [84]	China	PFS	31.0	63/63	SCC	NA	1	Male	Ser + CT/CT(321/167)	NR	0.50(0.39-0.64)
								Female	Ser + CT/CT(37/12)	NR	0.94(0.33-2.74)
ASTRUM-002/2026 [85]	China	PFS	23.4	62/61	non-SCC	NA	1	Male	Ser ± CT/CT(157/156)	0.58(0.44-0.76)	0.51(0.38-0.67)
								Female	Ser ± CT/CT(57/54)	1.03(0.64-1.69)	0.70(0.42-1.15)
ORIENT-11/2020 [86], 2021 [87], 2022 [88]	China	PFS	30.8	61/61	non-SCC	NA	1	Male	Sin + CT/CT(204/99)	0.57(0.43-0.77)	0.44(0.33-0.59)
								Female	Sin + CT/CT(62/32)	0.99(0.56-1.77)	0.64(0.38-1.08)
ORIENT-12/2021 [89]	China	PFS	12.9	64/62	SCC	NA	1	Male	Sin + CT/CT(163/164)	NR	0.53(0.41-0.68)
								Female	Sin + CT/CT(16/14)	NR	0.58(0.25-1.35)
ORIENT-3/2022 [90]	China	OS	NR	61/60	SCC	NA	2	Male	Sin/CT(136/122)	0.73(0.55-0.96)	NR
								Female	Sin/CT(9/13)	0.59(0.18-1.71)	NR
Study-104/2017 [91]	Muti	OS	12.5	64/64	SCC	NA	1	Male	Ipi + CT/CT(326/309)	0.85(0.71-1.02)	NR
								Female	Ipi + CT/CT(62/52)	1.33(0.84-2.11)	NR

Abbreviations: ICI, immune checkpoint inhibitor; Niv, nivolumab; Ipi, ipilimumab; Pem, pembrolizumab; Ate, atezolizumab; Cem, cemiplimab; Ave, avelumab; Dur, durvalumab; Tre, tremelimumab; Tis, tislelizumab; Cam, camrelizumab; Sug, sugemalimab; Tor, toripalimab; Ser, serplulimab; Sin, sintilimab; CT, chemotherapy; Bev, bevacizumab; SCC, squamous cell carcinoma; NSCLC, non-small cell lung cancer; OS, overall survival; PFS, progression-free survival; HR, hazard ratio; CI, confidence interval; Muti, multi-region; NA, not available; NR, not reported; A, paclitaxel plus carboplatin; B, nab-paclitaxel plus carboplatin.

**Fig 1:**
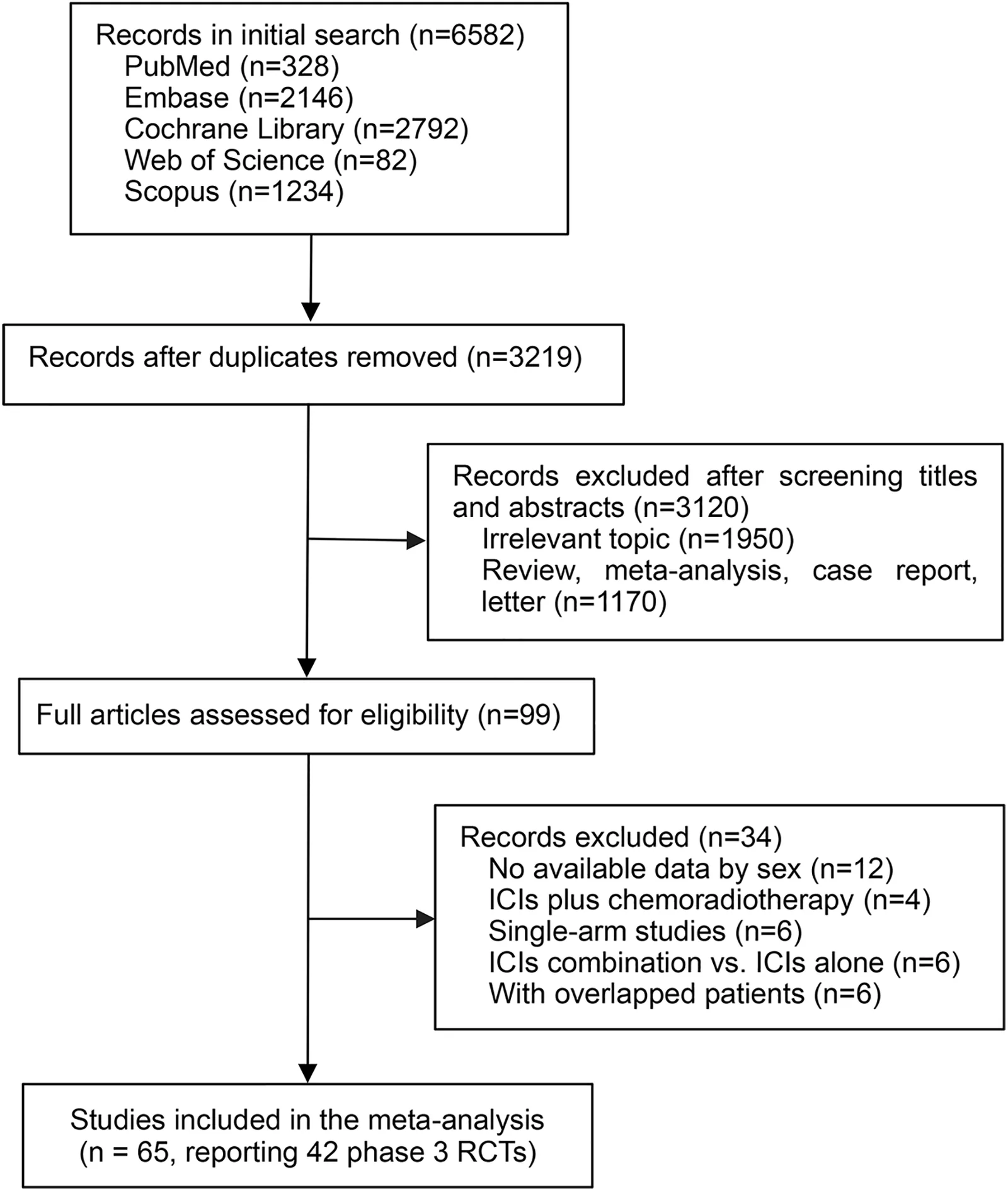
Literature search and selection. ICIs, immune checkpoint inhibitors.

### Effect of ICIs on OS and PFS by sex

Compared with chemotherapy, ICIs significantly improved both OS and PFS in both males (OS: HR = 0.74, 95% CI: 0.71–0.78, I^2^ = 40%; PFS: HR = 0.59, 95% CI: 0.54–0.64, I^2^ = 70%) and females (OS: HR = 0.83, 95% CI: 0.77–0.90, I^2^ = 35%; PFS: HR = 0.76, 95% CI: 0.66–0.86, I^2^ = 63%) (Figs 2 and 3). However, the pooled treatment-by-sex interaction HRs favored males significantly more than females for both OS (0.90, 95% CI: 0.84–0.97; P = 0.01) and PFS (0.82, 95% CI: 0.73–0.92; P = 0.0006), indicating a significantly greater treatment benefit from ICIs in males (Figs 2 and 3).

**Fig 2:**
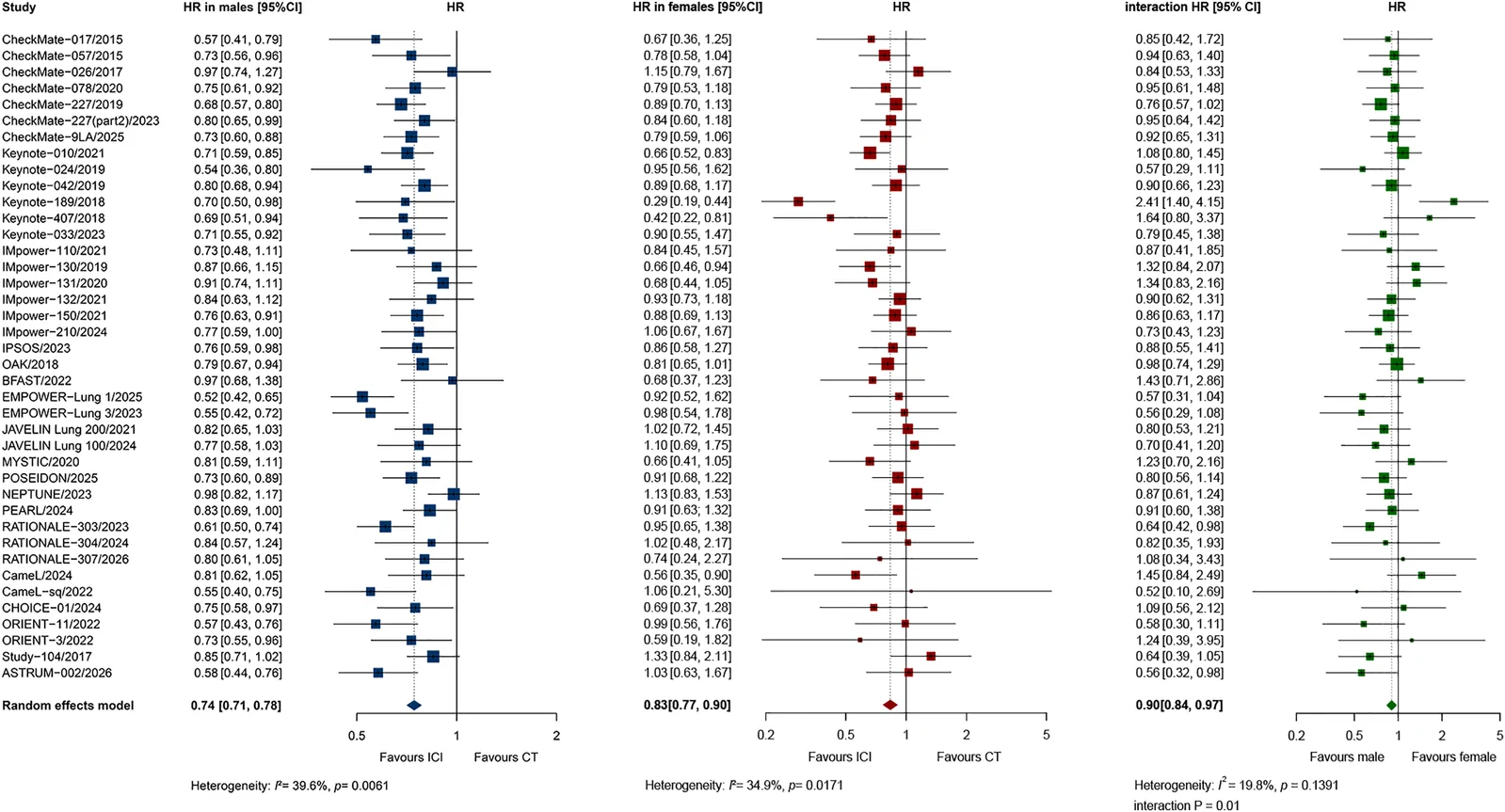
Forest plots of hazard ratios comparing overall survival between ICIs and CT and the interaction between treatment efficacy and sex. ICI, immune checkpoint inhibitor; CT, chemotherapy; HR, hazard ratio; CI, confidence interval.

**Fig 3:**
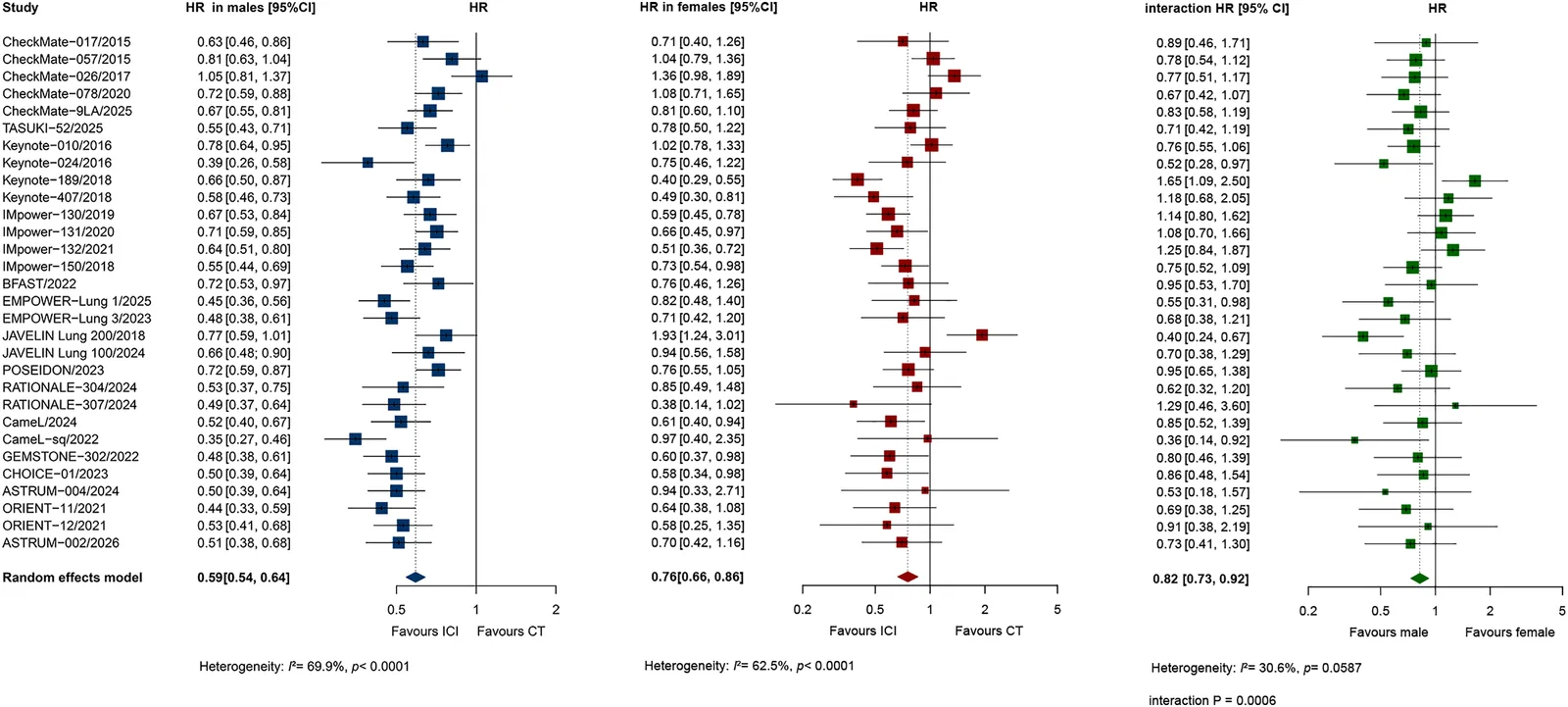
Forest plots of hazard ratios comparing progression-free survival between ICIs and CT and the interaction between treatment efficacy and sex. ICI, immune checkpoint inhibitor; CT, chemotherapy; HR, hazard ratio; CI, confidence interval.

### Subgroup analysis

In subgroup analyses for OS (Fig 4), ICIs significantly prolonged OS compared with chemotherapy across most subgroups for both sexes, including Asian and non‑Asian populations, non‑squamous cell carcinoma (non‑SCC), PD‑L1 expression ≥1%, 1L and ≥2L settings, and both ICI monotherapy (anti‑PD‑1 or anti‑PD‑L1) and ICI plus chemotherapy. Notably, significant OS improvements were observed among males but not among females in the squamous cell carcinoma (SCC), dual ICI therapy, and PD‑L1 expression ≥50% subgroups (all patients in the PD‑L1 ≥ 50% subgroup received ICI monotherapy). The pooled treatment-by-sex interaction HR was 0.73 (95% CI: 0.57–0.95; P = 0.02) for the PD-L1 ≥ 50%, 0.89 (95% CI: 0.80–0.98; P = 0.02) for the ICI monotherapy, 0.87 (95% CI: 0.76–0.99; P = 0.04) for anti-PD-1 monotherapy, and 0.82 (95% CI: 0.69–0.96; P = 0.03) for dual ICI therapy subgroups, indicating a significantly greater OS benefit for males in these subgroups. However, after the FDR correction, these sex differences did not retain statistical significance (P = 0.07, 0.07, 0.10, and 0.09, respectively).

**Fig 4:**
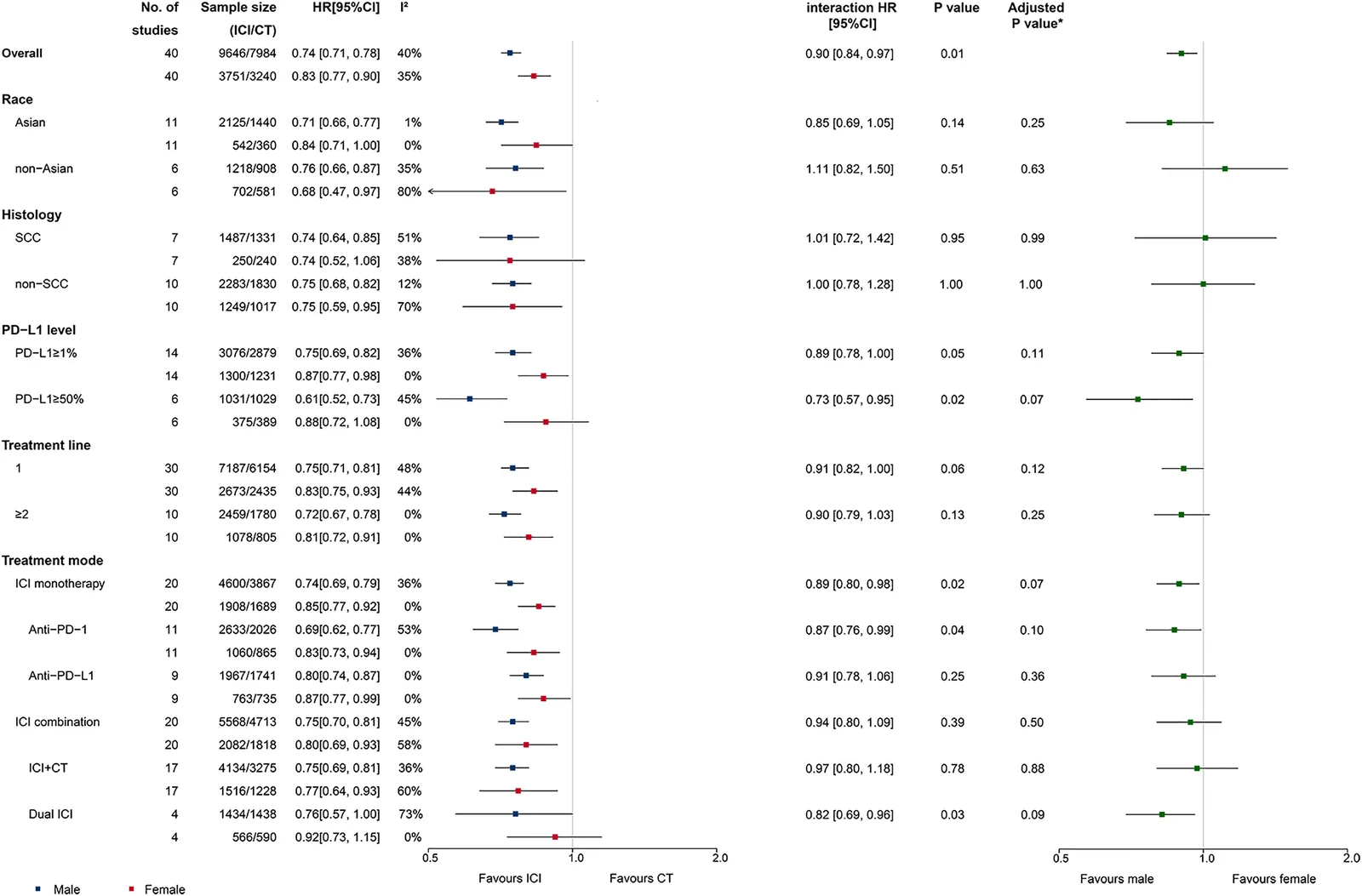
Subgroup analyses of overall survival according to sex and the interaction between treatment efficacy and sex. ICI, immune checkpoint inhibitor; CT, chemotherapy; SCC, squamous cell carcinoma; L, line; HR, hazard ratio; CI, confidence interval. * P-values after the False Discovery Rate correction.

In PFS subgroup analyses (Fig 5), ICIs significantly improved PFS in males across all subgroups; for females, significant PFS benefit was observed only in subgroups of Asian, SCC, non-SCC, 1L setting, and ICI plus chemotherapy. Significant sex-based difference in PFS benefit favoring males was observed in subgroups of Asian (P = 0.0007), PD-L1 expression ≥1% (P < 0.0001), PD-L1 expression ≥50% (P = 0.0008), 1L setting (P = 0.02), ≥ 2L setting (P = 0.04), ICI monotherapy (P < 0.0001), and anti-PD-1 monotherapy (P = 0.0001). After FDR correction, the sex differences remained significant in the Asian (P = 0.004), PD‑L1 ≥ 1% (P = 0.001), PD‑L1 ≥ 50% (P = 0.004), ICI monotherapy (P = 0.001), and anti‑PD‑1 monotherapy (P = 0.001) subgroups.

**Fig 5:**
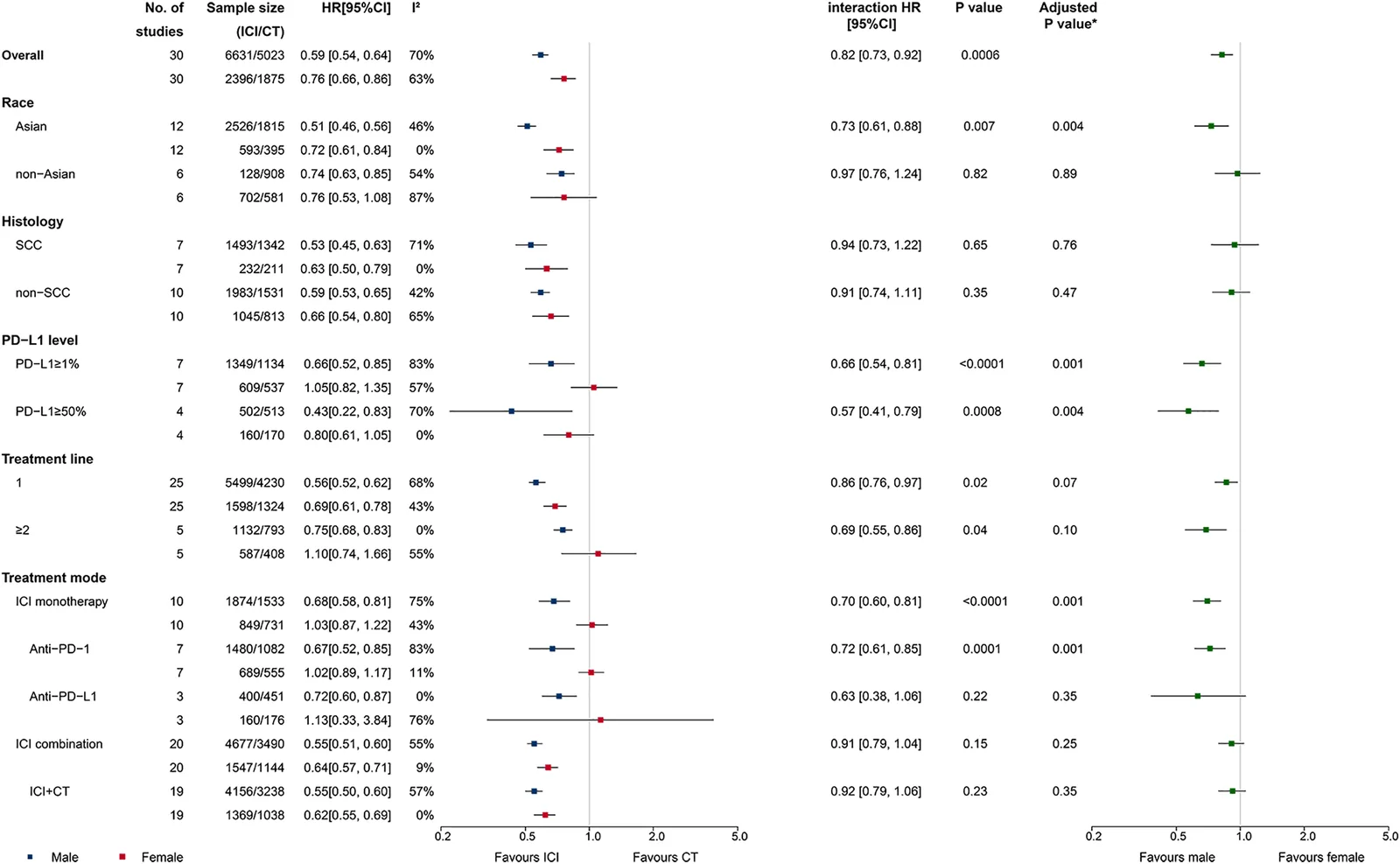
Subgroup analyses of progression-free survival according to sex and the interaction between treatment efficacy and sex. ICI, immune checkpoint inhibitor; CT, chemotherapy; SCC, squamous cell carcinoma; L, line; HR, hazard ratio; CI, confidence interval. * P-values after the False Discovery Rate correction.

Forest plots detailing the subgroup analysis results are shown in S1–S27 Figs (S3 File).

### The GRADE assessment

The overall quality of evidence (GRADE rating) for OS was moderate for both males and females. For PFS, the evidence quality was low for both sexes. In OS subgroup analyses, most evidence was rated as moderate quality. Exceptions with low-quality evidence were: SCC, and dual ICI subgroups for males; and non-Asian, non-SCC, and ICI plus chemotherapy combination subgroups for females. Detailed GRADE assessments are presented in S2 Table (S3 File).

### Meta-regression analysis

Multivariable meta‑regression analyses revealed that ICI type (P = 0.03) and race (P = 0.03) were significant sources of heterogeneity for OS and PFS, respectively, in males (S3 Table, S3 File). In females, ICI type (P = 0.04) and race (P = 0.04) were significantly associated with heterogeneity for OS, whereas PD‑L1 expression (P = 0.03) was significantly associated with heterogeneity for PFS (S3 Table, S3 File).

### Sensitivity analysis

The pooled HRs for males and females and the interaction HRs were robust, with no substantial changes in leave‑one‑out analyses (S28–S31 Figs, S3 File) or after excluding trials with ≤30 female patients in the intervention arms (CheckMate-017, IMpower-110, RATIONALE-307, CameL-sq, ORIENT-12, and ORIENT-3; S32 and S33 Figs, S3 File).

### Assessment of included trials and publication bias

Twelve trials [38,40,45,46,61,78,80,83,86,88] were assessed as having a low risk of bias. The remaining 30 trials were classified as having a high risk of bias, primarily due to their open-label design leading to lack of blinding of participants and personnel (S34 Fig, S3 File). Egger’s test results indicated no significant publication bias for either OS (males: P = 0.19; females: P = 0.65) or PFS (males: P = 0.13; females: P = 0.96). Corresponding funnel plots are shown in S35 Fig (S3 File).

## Discussion

This meta-analysis demonstrated that ICIs significantly prolonged both OS and PFS compared with chemotherapy in male and female patients with advanced NSCLC. Notably, male patients derived significantly greater survival benefits from ICIs than female patients. Subgroup analyses revealed that significant sex-based differences in OS and PFS benefits were observed among patients receiving ICI monotherapy (including anti-PD-1 monotherapy and those with PD-L1 expression ≥50%), but not among those treated with ICI plus chemotherapy.

The observed sex difference in treatment efficacy may be attributed to underlying biological mechanisms. Genomic and tumor microenvironment analyses reveal that female NSCLC patients exhibit greater T-cell dysfunction, elevated expression of inhibitory immune checkpoints, and a higher abundance of immunosuppressive cells compared to males [4]. Furthermore, clinical studies consistently report a lower prevalence of high PD-L1-expressing tumors in females compared to males [92–96]. Collectively, these factors suggest a reduced efficacy of ICI monotherapy in females. In addition, evidence indicates that females may have a higher response to chemotherapy than males [97, 98], which could further diminish the relative benefit of ICI monotherapy when compared with a chemotherapy control arm. However, the sex-based OS difference for ICI monotherapy (including anti‑PD‑1 monotherapy) lost statistical significance after FDR correction, despite the PFS difference remaining significant. It is therefore highly probable that the observed differences are at least partly driven by unbalanced baseline confounders For example, women generally have a lower smoking prevalence than men, and ICIs are less effective in smokers than in never‑smokers [2]. In addition, females were generally underrepresented in trials, particularly in major Asian trials utilizing anti‑PD‑1 agents, which may confound sex‑specific effect estimates. Therefore, this finding should be interpreted with caution and warrants validation in future RCTs specifically designed to investigate sex‑based differences.

The comparable efficacy of ICI plus chemotherapy between sexes may be explained by the ability of chemotherapy to enhance tumor antigenicity. Although female tumors may inherently possess weaker antigenicity, the addition of chemotherapy can increase tumor mutational burden [99], thereby promoting immune recognition and tumor cell elimination [100]. This effect might mitigate the underlying biological differences affecting ICI response in females.

In the CheckMate 227 trial [34], patients with PD-L1 < 1–49% derived lower OS benefit from nivolumab plus ipilimumab compared to those with PD-L1 ≥ 50% (HR = 0.94, 95% CI: 0.75–1.18 vs. HR = 0.70, 95% CI: 0.55–0.90). In the NEPTUNE trial [69], subgroup analysis suggested improved OS with durvalumab plus tremelimumab versus chemotherapy in patients with tumor mutational burden (TMB) ≥ 28 mut/Mb (HR = 0.49, 95% CI: 0.27–0.89) but not in those with TMB < 28 mut/Mb (HR = 1.04, 95% CI: 0.85–1.27). These results suggest better efficacy of dual ICI therapy in patients with high PD-L1 expression and high TMB, and provide explanations for our finding of greater OS benefit from dual ICI therapy in males than females. As noted above, high PD-L1-expressing tumors are less prevalent in females than in males. Moreover, male NSCLC patients often have a history of smoking and a high TMB. Nevertheless, similar to the findings for ICI monotherapy, the sex difference in OS benefit from dual ICI therapy was not significant after FDR correction. Thus, this finding also requires validation in future RCTs.

We also observed a significantly larger PFS benefit favoring males in the Asian population and among patients with PD‑L1 ≥ 1%, even after FDR correction, whereas no significant OS difference was found in these subgroups. Preclinical studies suggest that estrogen may upregulate PD‑L1 expression in lung cancer [101,102], which would theoretically favor ICI response in females. However, most patients with advanced NSCLC are elderly, a population in which estrogen levels decline markedly in females.

Previous meta-analyses examining sex differences in ICI efficacy across various cancers have yielded conflicting results [5–19], as summarized in S4 Table (S3 File). Four concluded males derived greater benefit [1,6,8,11 8], six found no significant difference [9,10,12,14], three reported better efficacy of ICI combination therapy in females [7,15,16], and two observed sex differences specific to melanoma (but not NSCLC) [5] or to ≥2L therapy (but not 1L) [17]. Several factors may contribute to this inconsistency. Many meta-analyses pooled data from diverse cancer types (e.g., NSCLC, SCLC, melanoma, genitourinary cancers) [5,6,9,10,13,17,18], which exhibit distinct tumor biologies and immunotherapy responses. NSCLC-focused meta-analyses [7,8,11,12,14,16,19] often included limited studies and did not incorporate recently updated long‑term survival data. These limitations compromise result stability and contribute to the divergent conclusions.

Our study has several strengths compared with prior meta-analyses. First, we exclusively focused on phase 3 RCTs comparing ICI with chemotherapy in *advanced* NSCLC, excluding other cancer types and early-stage disease. Second, we incorporated the latest evidence, including newly published trials and updated long-term survival data from existing studies. Third, we conducted extensive subgroup analyses based on race, histology, ICI class (anti-PD-1 vs. anti-PD-L1), treatment modality (monotherapy vs. combination), treatment line (1L vs. ≥ 2L), and PD-L1 expression level. These features enhance the stability and credibility of our findings. Crucially, our analysis reveals that females appear to derive less benefit than males from ICI monotherapy, even among those with PD‑L1 ≥ 50%, but exhibit comparable benefit from ICI plus chemotherapy. These findings may guide clinicians in developing individualized ICI treatment strategies for female patients with advanced NSCLC.

Nonetheless, our study has several limitations. First, because our analysis relied on data from published trials rather than individual patient data, it is not possible to fully isolate sex as an independent modifier from unbalanced baseline confounders (e.g., smoking status, histology, and PD‑L1 expression) within the cohorts. Furthermore, the underrepresentation of women in many trials may systematically bias sex‑specific effect estimates. Second, the sex‑based differences in OS in many subgroups became non‑significant after FDR correction, indicating a potential risk of false‑positive results. Finally, although the treatment‑by‑sex interaction for OS was statistically significant, the effect size was modest (HR ≈ 0.9). It should be recognized that in a large meta‑analysis such as ours, even minor effects can attain statistical significance. Therefore, our findings should be interpreted with due caution.

## Conclusions

Although ICIs are superior to chemotherapy in both sexes, the PFS benefit of ICI monotherapy appears to be attenuated in females relative to males. Given the imbalances in baseline confounders coupled with the underrepresentation of females in the included trials, these findings should be interpreted with caution and warrant confirmation in future RCTs designed specifically to investigate sex‑based differences.

## Supporting information

S1 File PRISMA 2020 checklist. (DOCX)

S2 File Llist of the excluded studies in Full-text assessing section. (DOCX)

S3 File Supplementary materials containing S1–S4 Tables and S1–S35 Figs. (DOCX)
