# Supplementary material for: Sex difference in survival benefits of immune checkpoint inhibitors in advanced non-small cell lung cancer: A meta-analysis
Source: PLoS One. 2026 Nov 3;21(11):e0361953. doi: 10.1371/journal.pone.0361953 (PMC13730348; doi:10.1371/journal.pone.0361953)
Supplement: Supplementary file 2 [file pone.0361953.s002.docx]

**List of the excluded studies in Full-text assessing section**

**1. No available data by sex (n=12)**

1) Zhong H, Sun S, Chen J, Wang Z, Zhao Y, Zhang G, et al. First-line penpulimab combined with paclitaxel and carboplatin for metastatic squamous non-small-cell lung cancer in China (AK105-302): a multicentre, randomised, double-blind, placebo-controlled phase 3 clinical trial. Lancet Respir Med. 2024;12(5):355-365.

2) Satouchi M, Nosaki K, Takahashi T, Nakagawa K, Aoe K, Kurata T, et al. First-line pembrolizumab vs chemotherapy in metastatic non-small-cell lung cancer: KEYNOTE-024 Japan subset. Cancer Sci. 2021;112(12):5000-5010.

3) Nishio M, Saito H, Goto K, Watanabe S, Sueoka-Aragane N, Okuma Y, et al. IMpower132: Atezolizumab plus platinum-based chemotherapy vs chemotherapy for advanced NSCLC in Japanese patients. Cancer Sci. 2021;112(4):1534-1544.

4) Horinouchi H, Nogami N, Saka H, Nishio M, Tokito T, Takahashi T, et al. Pembrolizumab plus pemetrexed-platinum for metastatic nonsquamous non-small-cell lung cancer: KEYNOTE-189 Japan Study. Cancer Sci. 2021;112(8):3255-3265.

5) Cheng Y, Zhou Q, Han B, Fan Y, Shan L, Chang J, et al. NEPTUNE China cohort: First-line durvalumab plus tremelimumab in Chinese patients with metastatic non-small-cell lung cancer. Lung Cancer. 2023;178:87-95.

6) Brahmer JR, Lee JS, Ciuleanu TE, Bernabe Caro R, Nishio M, Urban L, et al. Five-Year Survival Outcomes With Nivolumab Plus Ipilimumab Versus Chemotherapy as First-Line Treatment for Metastatic Non-Small-Cell Lung Cancer in CheckMate 227. J Clin Oncol. 2023;41(6):1200-1212.

7) Baramidze A, Makharadze T, Gogishvili M, Melkadze T, Giorgadze D, Penkov K, et al. Cemiplimab plus chemotherapy versus chemotherapy alone in non-small cell lung cancer with PD-L1 ≥ 1 %: A subgroup analysis from the EMPOWER-Lung 3 part 2 trial. Lung Cancer. 2024;193:107821.

8) Reck M, Rodríguez-Abreu D, Robinson AG, Hui R, Csőszi T, Fülöp A, et al. Five-Year Outcomes With Pembrolizumab Versus Chemotherapy for Metastatic Non-Small-Cell Lung Cancer With PD-L1 Tumor Proportion Score ≥ 50. J Clin Oncol. 2021;39(21):2339-2349.

9) Awad MM, Gadgeel SM, Borghaei H, Patnaik A, Yang JC, Powell SF, et al. Long-Term Overall Survival From KEYNOTE-021 Cohort G: Pemetrexed and Carboplatin With or Without Pembrolizumab as First-Line Therapy for Advanced Nonsquamous NSCLC. J Thorac Oncol. 2021;16(1):162-168.

10) Mazieres J, Rittmeyer A, Gadgeel S, Hida T, Gandara DR, Cortinovis DL, et al. Atezolizumab Versus Docetaxel in Pretreated Patients With NSCLC: Final Results From the Randomized Phase 2 POPLAR and Phase 3 OAK Clinical Trials. J Thorac Oncol. 2021;16(1):140-150.

11) Nosaki K, Saka H, Hosomi Y, Baas P, de Castro G Jr, Reck M, et al. Safety and efficacy of pembrolizumab monotherapy in elderly patients with PD-L1-positive advanced non-small-cell lung cancer: Pooled analysis from the KEYNOTE-010, KEYNOTE-024, and KEYNOTE-042 studies. Lung Cancer. 2019 ;135:188-195.

12) Cheng Y, Zhang L, Hu J, Wang D, Hu C, Zhou J, et al. Pembrolizumab Plus Chemotherapy for Chinese Patients With Metastatic Squamous NSCLC in KEYNOTE-407. JTO Clin Res Rep. 2021;2(10):100225.

**2. ICI plus chemoradiotherapy (n=4)**

1) Antonia SJ, Villegas A, Daniel D, Vicente D, Murakami S, Hui R, et al. Durvalumab after Chemoradiotherapy in Stage III Non-Small-Cell Lung Cancer. N Engl J Med. 2017;377(20):1919-1929.

2) Zhou Q, Chen M, Jiang O, Pan Y, Hu D, Lin Q, et al. Sugemalimab versus placebo after concurrent or sequential chemoradiotherapy in patients with locally advanced, unresectable, stage III non-small-cell lung cancer in China (GEMSTONE-301): interim results of a randomised, double-blind, multicentre, phase 3 trial. Lancet Oncol. 2022;23(2):209-219.

3) Wu YL, Wu L, Bi N, Cil T, Ge H, Zhu Z, et al. PACIFIC-5: a phase III clinical trial of consolidation durvalumab in patients with unresectable stage III NSCLC and no progression after concurrent or sequential chemoradiotherapy. J Hematol Oncol. 2025;18(1):111.

4) Bradley JD, Sugawara S, Lee KH, Ostoros G, Demirkazik A, Zemanova M, et al. Simultaneous Durvalumab and Platinum-Based Chemoradiotherapy in Unresectable Stage III Non-Small Cell Lung Cancer: The Phase III PACIFIC-2 Study. J Clin Oncol. 2025;43(33):3610-3621.

**3. Single-arm studies (n=6)**

1) Spigel DR, McCleod M, Jotte RM, Einhorn L, Horn L, Waterhouse DM, et al. Safety, Efficacy, and Patient-Reported Health-Related Quality of Life and Symptom Burden with Nivolumab in Patients with Advanced Non-Small Cell Lung Cancer, Including Patients Aged 70 Years or Older or with Poor Performance Status (CheckMate 153). J Thorac Oncol. 2019;14(9):1628-1639.

2) Ardizzoni A, Azevedo S, Rubio-Viqueira B, Rodriguez-Abreu D, Alatorre-Alexander J, Smit HJM, et al. Final results from TAIL: updated long-term efficacy of atezolizumab in a diverse population of patients with previously treated advanced non-small cell lung cancer. J Immunother Cancer. 2022;10(11):e005581.

3) Masuda T, Fujitaka K, Suzuki T, Hamai K, Matsumoto N, Matsumura M, et al. Phase 2 study of first-line pembrolizumab monotherapy in elderly patients with non-small-cell lung cancer expressing high PD-L1. Thorac Cancer. 2022;13(11):1611-1618.

4) Zhu W, Geng Q, Peng H, Jin Z, Li D, Pu X, et al. Efficacy and Safety of Low-Dose Nab-Paclitaxel Plus Tislelizumab in Elderly Patients With Previously Treated Metastatic Non-Small Cell Lung Cancer. Front Oncol. 2022;12:802467.

5) Seto T, Nosaki K, Shimokawa M, Toyozawa R, Sugawara S, Hayashi H, et al. Phase II study of atezolizumab with bevacizumab for non-squamous non-small cell lung cancer with high PD-L1 expression (@Be Study). J Immunother Cancer. 2022;10(2):e004025.

6) Borghaei H, Ciuleanu TE, Lee JS, Pluzanski A, Caro RB, Gutierrez M, et al. Long-term survival with first-line nivolumab plus ipilimumab in patients with advanced non-small-cell lung cancer: a pooled analysis. Ann Oncol. 2023;34(2):173-185.

**4. ICI combination vs ICI alone (n=6)**

1) Boyer M, Şendur MAN, Rodríguez-Abreu D, Park K, Lee DH, Çiçin I, et al. Pembrolizumab Plus Ipilimumab or Placebo for Metastatic Non-Small-Cell Lung Cancer With PD-L1 Tumor Proportion Score ≥ 50%: Randomized, Double-Blind Phase III KEYNOTE-598 Study. J Clin Oncol. 2021;39(21):2327-2338.

2) Shiraishi Y, Kishimoto J, Sugawara S, Mizutani H, Daga H, Azuma K, et al. Atezolizumab and Platinum Plus Pemetrexed With or Without Bevacizumab for Metastatic Nonsquamous Non-Small Cell Lung Cancer: A Phase 3 Randomized Clinical Trial. JAMA Oncol. 2024;10(3):315-324.

3) Shiraishi Y, Nomura S, Sugawara S, Horinouchi H, Yoneshima Y, Hayashi H, et al. Comparison of platinum combination chemotherapy plus pembrolizumab versus platinum combination chemotherapy plus nivolumab-ipilimumab for treatment-naive advanced non-small-cell lung cancer in Japan (JCOG2007): an open-label, multicentre, randomised, phase 3 trial. Lancet Respir Med. 2024;12(11):877-887.

4) Zhong H, Wang J, Yang R, Luo Y, Zuo W, Zhang W, et al. Benmelstobart plus anlotinib versus pembrolizumab as first-line treatment for PD-L1-positive, advanced non-small-cell lung cancer (CAMPASS): a blinded, randomised, controlled, phase 3 trial. Lancet Oncol. 2026;27(4):419-431.

5) Xiong A, Wang L, Chen J, Wu L, Liu B, Yao J, et al. Ivonescimab versus pembrolizumab for PD-L1-positive non-small cell lung cancer (HARMONi-2): a randomised, double-blind, phase 3 study in China. Lancet. 2025;405(10481):839-849.

6) Chen Z, Yang F, Jiang Z, Sun L, Wu L, Han Z, et al. Ivonescimab plus chemotherapy versus tislelizumab plus chemotherapy as first-line treatment for advanced squamous non-small-cell lung cancer (HARMONi-6): a randomised, double-blind, phase 3 trial. Lancet. 2025;406(10515):2078-2088.

**5. With overlapped patients (n=6)**

1) Zhou C, Chen G, Huang Y, Zhou J, Lin L, Feng J, et al. Camrelizumab Plus Carboplatin and Pemetrexed as First-Line Treatment for Advanced Nonsquamous NSCLC: Extended Follow-Up of CameL Phase 3 Trial. J Thorac Oncol. 2023;18(5):628-639.

2) Reck M, Ciuleanu TE, Schenker M, Bordenave S, Cobo M, Juan-Vidal O, et al. Five-year outcomes with first-line nivolumab plus ipilimumab with 2 cycles of chemotherapy versus 4 cycles of chemotherapy alone in patients with metastatic non-small cell lung cancer in the randomized CheckMate 9LA trial. Eur J Cancer. 2024;211:114296.

3) Özgüroğlu M, Kilickap S, Sezer A, Gümüş M, Bondarenko I, Gogishvili M, et al. First-line cemiplimab monotherapy and continued cemiplimab beyond progression plus chemotherapy for advanced non-small-cell lung cancer with PD-L1 50% or more (EMPOWER-Lung 1): 35-month follow-up from a mutlicentre, open-label, randomised, phase 3 trial. Lancet Oncol. 2023;24(9):989-1001.

4) Herbst RS, Garon EB, Kim DW, Cho BC, Perez-Gracia JL, Han JY, et al. Long-Term Outcomes and Retreatment Among Patients With Previously Treated, Programmed Death-Ligand 1‒Positive, Advanced Non‒Small-Cell Lung Cancer in the KEYNOTE-010 Study. J Clin Oncol. 2020;38(14):1580-1590.

5) Rittmeyer A, Barlesi F, Waterkamp D, Park K, Ciardiello F, et al. Atezolizumab versus docetaxel in patients with previously treated non-small-cell lung cancer (OAK): a phase 3, open-label, multicentre randomised controlled trial. Lancet. 2017;389(10066):255-265.

6) Hellmann MD, Ciuleanu TE, Pluzanski A, Lee JS, Otterson GA, Audigier-Valette C, et al. Nivolumab plus Ipilimumab in Lung Cancer with a High Tumor Mutational Burden. N Engl J Med. 2018;378(22):2093-2104.
