# Supplementary material for: Sex difference in survival benefits of immune checkpoint inhibitors in advanced non-small cell lung cancer: A meta-analysis
Source: PLoS One. 2026 Nov 3;21(11):e0361953. doi: 10.1371/journal.pone.0361953 (PMC13730348; doi:10.1371/journal.pone.0361953)
Supplement: Supplementary file 3 [file pone.0361953.s003.docx]

**S1 Table** Search strategy

**a:** Search strategy in PubMed

| # | Query |
| --- | --- |
| #1 | "Carcinoma, Non-Small-Cell Lung"[mh] |
| #2 | "nsclc"[Title/Abstract] |
| #3 | "lung cancer*"[Title/Abstract] OR "lung carcinoma*"[Title/Abstract] OR "lung neoplasm*"[Title/Abstract] OR "lung tumor*"[Title/Abstract] OR "lung tumour*"[Title/Abstract] |
| #4 | "non small cell*"[Title/Abstract] OR "nonsmall cell*"[Title/Abstract] |
| #5 | #3 AND #4 |
| #6 | #1 OR #2 OR #5 |
| #7 | "Immunotherapy"[Mesh] |
| #8 | "Immune Checkpoint Inhibitors"[Mesh] |
| #9 | "Immunotherapies"[Title/Abstract] OR "programmed death ligand 1"[Title/Abstract] OR "PD-L1"[Title/Abstract] OR "programmed death 1"[Title/Abstract] OR "PD-1"[Title/Abstract] OR "anti programmed death ligand 1"[Title/Abstract] OR "Anti-PD-L1"[Title/Abstract] OR "anti programmed death 1"[Title/Abstract] OR "Anti-PD-1"[Title/Abstract] OR "Anti-CTLA-4"[Title/Abstract] OR "Atezolizumab"[Title/Abstract] OR "Durvalumab"[Title/Abstract] OR "Nivolumab"[Title/Abstract] OR "Pembrolizumab"[Title/Abstract] OR "Camrelizumab"[Title/Abstract] OR "Sintilimab"[Title/Abstract] OR "Cemiplimab"[Title/Abstract] OR "Tislelizumab"[Title/Abstract] OR "Ipilimumab"[Title/Abstract] OR "Avelumab"[Title/Abstract] OR "Tremelimumab"[Title/Abstract] OR "Sugemalimab"[Title/Abstract] OR "Serplulimab"[Title/Abstract] OR "Toripalimab"[Title/Abstract] |
| #10 | #7 OR #8 OR #9 |
| #11 | "Advanced"[Title/Abstract] OR "stage 4"[Title/Abstract] OR "stage iv"[Title/Abstract] OR "metasta*"[Title/Abstract] |
| #12 | ("randomized controlled trial"[Publication Type]) OR (((RCT[Title/Abstract]) OR ("randomized controlled trial"[Title/Abstract]))) |
| #13 | #6 AND #10 AND #11 AND #12 |

**b:** Search strategy in Embase

| # | Query |
| --- | --- |
| #1 | ‘non small cell lung cancer’/exp |
| #2 | ’nsclc’:ab,ti |
| #3 | 'non small cell*':ab,ti OR 'nonsmall cell*':ab,ti |
| #4 | 'lung cancer*':ab,ti OR 'lung carcinoma*':ab,ti OR 'lung neoplasm*':ab,ti OR 'lung tumor*':ab,ti OR 'lung tumour*':ab,ti |
| #5 | #3 AND #4 |
| #6 | #1 OR #2 OR #5 |
| #7 | ‘immunotherapy'/exp |
| #8 | 'immune checkpoint inhibitor'/exp |
| #9 | ‘programmed death ligand 1’:ab,ti OR ‘PD-L1’:ab,ti OR ‘programmed death 1’:ab,ti OR ‘PD-1’:ab,ti OR ‘anti-programmed death ligand 1’:ab,ti OR ‘anti–PD-L1’:ab,ti OR ‘anti-programmed death 1’:ab,ti OR ‘anti–PD-1’:ab,ti OR ‘atezolizumab’:ab,ti OR ‘durvalumab’:ab,ti OR ‘nivolumab’:ab,ti OR ‘pembrolizumab’:ab,ti OR ‘camrelizumab’:ab,ti OR ‘sintilimab’:ab,ti OR ‘cemiplimab’:ab,ti OR ‘avelumab’:ab,ti OR ‘tislelizumab’:ab,ti OR ‘anti–CTLA-4’:ab,ti OR ‘ipilimumab’:ab,ti OR ‘tremelimumab’:ab,ti OR ‘sugemalimab’:ab,ti OR ‘serplulimab’:ab,ti OR ‘toripalimab’:ab,ti |
| #10 | #7 OR #8 OR #9 |
| #11 | 'advanced':ab,ti OR 'stage 4':ab,ti OR 'stage iv':ab,ti OR 'metasta*':ab,ti |
| #12 | 'controlled clinical trial'/de OR 'randomized controlled trial'/de |
| #13 | #6 AND #10 AND #11 AND #12 |

**c:** Search strategy in Cochrane Library

| # | Query |
| --- | --- |
| #1 | MeSH descriptor: [Carcinoma, Non-Small-Cell Lung] explode all trees |
| #2 | nsclc |
| #3 | lung cancer* |
| #4 | lung carcinom* |
| #5 | lung neoplasm* |
| #6 | lung tumor* |
| #7 | lung tumour* |
| #8 | non small cell* |
| #9 | nonsmall cell* |
| #10 | #3 OR #4 OR #5 OR #6 OR #7 |
| #11 | #8 OR #9 |
| #12 | #10 AND #11 |
| #13 | #1 OR #2 OR #12 |
| #14 | MeSH descriptor: [Immunotherapy] explode all trees |
| #15 | MeSH descriptor: [Immune Checkpoint Inhibitors] explode all trees |
| #16 | (programmed death ligand 1 OR PD-L1 OR programmed death 1 OR PD-1 OR anti-programmed death ligand 1 OR anti–PD-L1 OR anti-programmed death 1 OR anti–PD-1 OR atezolizumab OR durvalumab OR nivolumab OR pembrolizumab OR camrelizumab OR sintilimab OR cemiplimab OR avelumab OR tislelizumab OR anti–CTLA-4 OR ipilimumab OR tremelimumab OR sugemalimab OR serplulimab OR toripalimab):ti,ab,kw |
| #17 | #14 OR #15 OR #16 |
| #18 | (advanced OR "stage 4" OR "stage IV" OR metasta*):ti,ab,kw |
| #19 | #13 AND #17 AND #18 |

**d:** Search strategy in Web of Science

| # | Query |
| --- | --- |
| #1 | TS=("lung cancer" OR "non-small cell lung cancer" OR NSCLC OR ((lung OR pulmon*) AND (neoplas* OR cancer OR carcinoma* OR tumour* or tumor*) AND ((“non small cell*”) OR (“nonsmall cell*”)))) |
| #2 | TS=(“programmed death ligand 1” OR “PD-L1” OR “programmed death 1” OR “PD-1” OR “anti-programmed death ligand 1” OR “anti–PD-L1” OR “anti-programmed death 1” OR “anti–PD-1” OR “atezolizumab” OR “durvalumab” OR “nivolumab” OR “pembrolizumab” OR “camrelizumab” OR “sintilimab” OR “tislelizumab” OR “cemiplimab” OR “avelumab” OR “tislelizumab” OR “anti–CTLA-4” OR “ipilimumab” OR “tremelimumab” OR “sugemalimab” OR “serplulimab” OR “toripalimab”) |
| #3 | TS=("advanced" OR "stage 4" OR "stage iv" OR "metasta*") |
| #4 | TS=("randomized controlled trial" OR "controlled clinical trial") |
| #5 | #1 AND #2 AND #3 AND #4 |

**e:** Search strategy in Scopus

| # | Query |
| --- | --- |
| #1 | TITLE-ABS-KEY ({Carcinoma, Non-Small-Cell Lung}) |
| #2 | TITLE-ABS-KEY (nsclc) |
| #3 | #1 OR #2 |
| #4 | TITLE-ABS-KEY ({Immunotherapy} OR {Immune Checkpoint Inhibitors}) |
| #5 | TITLE-ABS ("Immunotherapies" OR "programmed death ligand 1" OR "PD-L1" OR "programmed death 1" OR "PD-1" OR "anti programmed death ligand 1" OR "Anti-PD-L1" OR "anti programmed death 1" OR "Anti-PD-1" OR "Anti-CTLA-4" OR "Atezolizumab" OR "Durvalumab" OR "Nivolumab" OR "Pembrolizumab" OR "Camrelizumab" OR "Sintilimab" OR "Cemiplimab" OR "Tislelizumab" OR "Ipilimumab" OR "Avelumab" OR "Tremelimumab" OR "Sugemalimab" OR "Serplulimab" OR "Toripalimab") |
| #6 | #4 OR #5 |
| #7 | TITLE-ABS ("Advanced" OR "stage 4" OR "stage iv" OR "metasta*") |
| #8 | TITLE-ABS-KEY ("randomized controlled trial") |
| #9 | TITLE-ABS-KEY ("controlled clinical trial") |
| #10 | #8 OR #9 |
| #11 | #3 AND #6 AND #7 AND #10 |

**S2 Table** GRADE assessment

| Outcome | No. of studies /patients | Risk of bias | Inconsistency | Indirectness | Imprecision | Publication bias | Large effect size | Dose effect | Other consideration | GRADE of evidence |
| --- | --- | --- | --- | --- | --- | --- | --- | --- | --- | --- |
| **Male** | | | | | | | | | | |
| OS | 38/16977 | Not serious | Not serious | Not serious | Not serious | Not serious | Not present | Not present | Not present | Moderate |
| PFS | 28/10571 | Not serious | Serious (I^2^=70%) | Not serious | Not serious | Not serious | Not present | Not present | Not present | Low |
| Subgroup of OS | | | | | | | | | | |
| Asian | 9/2912 | Not serious | Not serious | Not serious | Not serious | Not tested | Not present | Not present | Not present | Moderate |
| non-Asian | 6/2126 | Not serious | Not serious | Not serious | Not serious | Not tested | Not present | Not present | Not present | Moderate |
| SCC | 6/2478 | Not serious | Serious (I^2^=59%) | Not serious | Not serious | Not tested | Not present | Not present | Not present | Low |
| non-SCC | 9/3800 | Not serious | Not serious | Not serious | Not serious | Not tested | Not present | Not present | Not present | Moderate |
| PD-L1 ≥1% | 14/5955 | Not serious | Not serious | Not serious | Not serious | Not tested | Not present | Not present | Not present | Moderate |
| PD-L1 ≥50% | 5/1981 | Not serious | Not serious | Not serious | Not serious | Not tested | Not present | Not present | Not present | Moderate |
| 1L setting | 28/12688 | Not serious | Not serious | Not serious | Not serious | Not tested | Not present | Not present | Not present | Moderate |
| ≥2L setting | 10/4239 | Not serious | Not serious | Not serious | Not serious | Not tested | Not present | Not present | Not present | Moderate |
| ICIs monotherapy | 21/8968 | Not serious | Not serious | Not serious | Not serious | Not tested | Not present | Not present | Not present | Moderate |
| Anti-PD-1 | 11/4659 | Not serious | Not serious | Not serious | Not serious | Not tested | Not present | Not present | Not present | Moderate |
| Anti-PD-L1 | 10/4208 | Not serious | Not serious | Not serious | Not serious | Not tested | Not present | Not present | Not present | Moderate |
| ICIs combination | 18/8427 | Not serious | Not serious | Not serious | Not serious | Not tested | Not present | Not present | Not present | Moderate |
| ICIs +CT | 14/6255 | Not serious | Not serious | Not serious | Not serious | Not tested | Not present | Not present | Not present | Moderate |
| Dual ICIs | 4/2872 | Not serious | Serious (I^2^=74%) | Not serious | Not serious | Not tested | Not present | Not present | Not present | Low |
| Subgroup of PFS | | | | | | | | | | |
| Asian | 11/4028 | Not serious | Serious (I^2^=51%) | Not serious | Not serious | Not tested | Not present | Not present | Not present | Low |
| non-Asian | 6/2126 | Not serious | Serious (I^2^=57%) | Not serious | Not serious | Not tested | Not present | Not present | Not present | Low |
| SCC | 7/2835 | Not serious | Serious (I^2^=71%) | Not serious | Not serious | Not tested | Not present | Not present | Not present | Low |
| non-SCC | 9/3201 | Not serious | Not serious | Not serious | Not serious | Not tested | Not present | Not present | Not present | Moderate |
| PD-L1 ≥1% | 7/2483 | Not serious | Serious (I^2^=82%) | Not serious | Not serious | Not tested | Not present | Not present | Not present | Low |
| PD-L1 ≥50% | 3/936 | Not serious | Serious (I^2^=60%) | Not serious | Serious (only 3 studies) | Not tested | Not present | Not present | Not present | Very low |
| 1L setting | 23/8646 | Not serious | Serious (I^2^=67%) | Not serious | Not serious | Not tested | Not present | Not present | Not present | Low |
| ≥2L setting | 5/1925 | Not serious | Not serious | Not serious | Not serious | Not tested | Not present | Not present | Not present | Moderate |
| ICIs monotherapy | 10/3407 | Not serious | Serious (I^2^=75%) | Not serious | Not serious | Not tested | Not present | Not present | Not present | Low |
| Anti-PD-1 | 7/2562 | Not serious | Serious (I^2^=83%) | Not serious | Not serious | Not tested | Not present | Not present | Not present | Low |
| Anti-PD-L1 | 3/851 | Not serious | Not serious | Not serious | Serious (only 3 studies) | Not tested | Not present | Not present | Not present | Low |
| ICIs +CT | 17/6580 | Not serious | Serious (I^2^=50%) | Not serious | Not serious | Not tested | Not present | Not present | Not present | Low |
| **Female** | | | | | | | | | | |
| OS | 38/6850 | Not serious | Not serious | Not serious | Not serious | Not serious | Not present | Not present | Not present | Moderate |
| PFS | 28/3917 | Not serious | Serious (I^2^=65%) | Not serious | Not serious | Not serious | Not present | Not present | Not present | Low |
| Subgroup of OS | | | | | | | | | | |
| Asian | 9/761 | Not serious | Not serious | Not serious | Not serious | Not tested | Not present | Not present | Not present | Moderate |
| non-Asian | 6/1283 | Not serious | Serious (I^2^=80%) | Not serious | Not serious | Not tested | Not present | Not present | Not present | Low |
| SCC | 6/460 | Not serious | Not serious | Not serious | Not serious | Not tested | Not present | Not present | Not present | Moderate |
| non-SCC | 9/2155 | Not serious | Serious (I^2^=72%) | Not serious | Not serious | Not tested | Not present | Not present | Not present | Low |
| PD-L1 ≥1% | 14/2531 | Not serious | Not serious | Not serious | Not serious | Not tested | Not present | Not present | Not present | Moderate |
| PD-L1 ≥50% | 5/733 | Not serious | Not serious | Not serious | Not serious | Not tested | Not present | Not present | Not present | Moderate |
| 1L setting | 28/4967 | Not serious | Not serious | Not serious | Not serious | Not tested | Not present | Not present | Not present | Moderate |
| ≥2L setting | 10/1883 | Not serious | Not serious | Not serious | Not serious | Not tested | Not present | Not present | Not present | Moderate |
| ICIs monotherapy | 21/3597 | Not serious | Not serious | Not serious | Not serious | Not tested | Not present | Not present | Not present | Moderate |
| Anti-PD-1 | 11/1925 | Not serious | Not serious | Not serious | Not serious | Not tested | Not present | Not present | Not present | Moderate |
| Anti-PD-L1 | 10/1672 | Not serious | Not serious | Not serious | Not serious | Not tested | Not present | Not present | Not present | Moderate |
| ICIs combination | 18/3585 | Not serious | Serious (I^2^=62%) | Not serious | Not serious | Not tested | Not present | Not present | Not present | Low |
| ICIs +CT | 14/2429 | Not serious | Serious (I^2^=66%) | Not serious | Not serious | Not tested | Not present | Not present | Not present | Low |
| Dual ICIs | 4/1156 | Not serious | Not serious | Not serious | Not serious | Not tested | Not present | Not present | Not present | Moderate |
| Subgroup of PFS | | | | | | | | | | |
| Asian | 11/877 | Not serious | Not serious | Not serious | Not serious | Not tested | Not present | Not present | Not present | Moderate |
| non-Asian | 6/1283 | Not serious | Serious (I^2^=87%) | Not serious | Not serious | Not tested | Not present | Not present | Not present | Low |
| SCC | 7/443 | Not serious | Not serious | Not serious | Not serious | Not tested | Not present | Not present | Not present | Moderate |
| non-SCC | 9/1747 | Not serious | Serious (I^2^=69%) | Not serious | Not serious | Not tested | Not present | Not present | Not present | Low |
| PD-L1 ≥1% | 7/1146 | Not serious | Serious (I^2^=57%) | Not serious | Not serious | Not tested | Not present | Not present | Not present | Low |
| PD-L1 ≥50% | 3/299 | Not serious | Not serious | Not serious | Serious (only 3 studies) | Not tested | Not present | Not present | Not present | Low |
| 1L setting | 23/2922 | Not serious | Not serious | Not serious | Not serious | Not tested | Not present | Not present | Not present | Moderate |
| ≥2L setting | 5/995 | Not serious | Serious (I^2^=55%) | Not serious | Not serious | Not tested | Not present | Not present | Not present | Low |
| ICIs monotherapy | 10/1580 | Not serious | Not serious | Not serious | Not serious | Not tested | Not present | Not present | Not present | Moderate |
| Anti-PD-1 | 7/1244 | Not serious | Not serious | Not serious | Not serious | Not tested | Not present | Not present | Not present | Moderate |
| Anti-PD-L1 | 3/336 | Not serious | Serious (I^2^=76%) | Not serious | Serious (only 3 studies) | Not tested | Not present | Not present | Not present | Very low |
| ICIs +CT | 17/2122 | Not serious | Not serious | Not serious | Not serious | Not tested | Not present | Not present | Not present | Moderate |

Abbreviations: GRADE, Grading of Recommendations, Assessment, Development and Evaluations; OS, overall survival; PFS, progression-free survival; SCC, squamous cell carcinoma; ICIs, immune checkpoint inhibitors; L, line; RCTs, randomized controlled trials.

**S3 Table** Meta-regression analysis

| Group | No. of studies | HR [95%CI] | Meta-regression (Univariate) | | Meta-regression (Multivariable) | |
| --- | --- | --- | --- | --- | --- | --- |
|  |  |  | β [95%CI] | P value | β [95%CI] | P value |
| **OS (male)** | | | | | | |
| Sample size | | | | | | |
| <100 | 0 |  |  |  |  |  |
| ≥100 | 40 |  |  |  |  |  |
| Risk bias | | | | | | |
| High | 31 | 0.77 [0.73, 0.81] |  |  |  |  |
| Low or unclear | 9 | 0.67 [0.60, 0.75] | -0.13 [-0.25, -0.02] | 0.03 | -0.11 [-0.26, 0.04] | 0.15 |
| Race | | | | | | |
| Asian | 11 | 0.71 [0.66, 0.77] |  |  |  |  |
| Non-Asian | 6 | 0.76 [0.66, 0.87] | 0.07 [-0.10, 0.24] | 0.44 | 0.01 [-0.17, 0.20] | 0.90 |
| Mixed | 23 | 0.75 [0.71, 0.81] | 0.06 [-0.06, 0.18] | 0.31 | -0.03 [-0.18, 0.11] | 0.65 |
| Histological type | | | | | | |
| SCC | 7 | 0.74 [0.64, 0.85] |  |  |  |  |
| Non-SCC | 9 | 0.74 [0.67, 0.81] | -0.01 [-0.18, 0.16] | 0.87 | -0.06 [-0.24, 0.13] | 0.55 |
| Mixed | 24 | 0.75 [0.70, 0.79] | 0.00 [-0.14, 0.14] | 1.00 | -0.03 [-0.19, 0.13] | 0.69 |
| PD-L1 status | | | | | | |
| ≥1% | 11 | 0.74 [0.67, 0.83] |  |  |  |  |
| <1% or NR | 29 | 0.75 [0.70, 0.79] | 0.00 [-0.11, 0.11] | 0.99 | -0.06 [-0.23, 0.11] | 0.51 |
| Treatment mode | | | | | | |
| ICI monotherapy | 20 | 0.74 [0.69, 0.79] |  |  |  |  |
| ICI combination | 19 | 0.75 [0.69, 0.81] | 0.02 [-0.08, 0.12] | 0.72 | 0.08 [-0.15, 0.32] | 0.50 |
| Mixed | 1 | 0.73 [0.60, 0.89] | -0.01 [-0.30, 0.28] | 0.94 | -0.02 [-0.40, 0.36] | 0.92 |
| ICI drug | | | | | | |
| Anti-PD-1 | 22 | 0.69 [0.64, 0.74] |  |  |  |  |
| Anti-PD-L1 | 13 | 0.81 [0.76, 0.87] | 0.16 [0.06, 0.26] | 0.002 | 0.15 [0.02, 0.29] | 0.03 |
| Others | 5 | 0.79 [0.69, 0.90] | 0.13 [0.01, 0.25] | 0.04 | 0.12 [-0.08, 0.32] | 0.25 |
| Treatment line | | | | | | |
| 1 line | 30 | 0.75 [0.71, 0.80] |  |  |  |  |
| ≥2 line | 10 | 0.72 [0.67, 0.78] | -0.04 [-0.16, 0.07] | 0.43 | 0.01 [-0.17, 0.18] | 0.93 |
| **PFS (male)** | | | | | | |
| Sample size | | | | | | |
| <100 | 0 |  |  |  |  |  |
| ≥100 | 30 |  |  |  |  |  |
| Risk bias | | | | | | |
| High | 18 | 0.64 [0.58, 0.71] |  |  |  |  |
| Low or unclear | 12 | 0.52 [0.47, 0.59] | -0.20 [-0.36, -0.05] | 0.01 | -0.03 [-0.25, 0.18] | 0.77 |
| Race | | | | | | |
| Asian | 12 | 0.51 [0.46, 0.57] |  |  |  |  |
| Non-Asian | 6 | 0.74 [0.63, 0.85] | 0.37 [0.17, 0.56] | <0.001 | 0.28 [0.03, 0.53] | 0.03 |
| Mixed | 12 | 0.62 [0.55, 0.69] | 0.19 [0.03, 0.35] | 0.02 | 0.06 [-0.16, 0.27] | 0.60 |
| Histological type | | | | | | |
| SCC | 7 | 0.53 [0.45, 0.63] |  |  |  |  |
| Non-SCC | 10 | 0.59 [0.53, 0.65] | 0.09 [-0.14, 0.31] | 0.44 | 0.01 [-0.18, 0.21] | 0.88 |
| Mixed | 13 | 0.63 [0.54, 0.73] | 0.17 [-0.05, 0.38] | 0.12 | 0.05 [-0.21, 0.30] | 0.73 |
| PD-L1 status | | | | | | |
| ≥1% | 6 | 0.65 [0.49, 0.87] |  |  |  |  |
| < 1% or NR | 24 | 0.58 [0.53, 0.63] | -0.14 [-0.35, 0.07] | 0.19 | -0.03 [-0.33, 0.28] | 0.87 |
| Treatment mode | | | | | | |
| ICI monotherapy | 10 | 0.69 [0.58, 0.81] |  |  |  |  |
| ICI combination | 19 | 0.55 [0.50, 0.59] | -0.24 [-0.40, -0.08] | 0.004 | -0.02 [-0.44, 0.40] | 0.93 |
| Mixed | 1 | 0.72 [0.59,0.87] | 0.04 [-0.36, 0.44] | 0.84 | 0.28 [-0.44, 1.00] | 0.45 |
| ICI drug | | | | | | |
| Anti-PD-1 | 20 | 0.56 [0.50, 0.63] |  |  |  |  |
| Anti-PD-L1 | 8 | 0.64 [0.58, 0.71] | 0.13 [-0.06, 0.32] | 0.17 | 0.12 [-0.09, 0.34] | 0.27 |
| Others | 2 | 0.69 [0.61, 0.80] | 0.21 [-0.10, 0.53] | 0.19 | -0.02 [-0.57, 0.52] | 0.94 |
| Treatment line | | | | | | |
| 1 line | 25 | 0.56 [0.52, 0.62] |  |  |  |  |
| ≥2 line | 5 | 0.76 [0.68, 0.84] | 0.28 [0.08, 0.48] | 0.005 | 0.22 [-0.07, 0.50] | 0.13 |
| **OS (female)** |  |  |  |  |  |  |
| Sample size |  |  |  |  |  |  |
| <100 | 10 | 0.87 [0.71, 1.08] |  |  |  |  |
| ≥100 | 30 | 0.83 [0.76, 0.91] | -0.04 [-0.29, 0.21] | 0.74 | -0.06 [-0.33, 0.22] | 0.69 |
| Risk bias |  |  |  |  |  |  |
| High | 31 | 0.87 [0.81, 0.92] |  |  |  |  |
| Low or unclear | 9 | 0.73 [0.51, 1.03] | -0.21 [-0.40, -0.02] | 0.03 | -0.16 [-0.42, 0.09] | 0.21 |
| Race |  |  |  |  |  |  |
| Asian | 11 | 0.84 [0.71, 1.00] |  |  |  |  |
| Non-Asian | 6 | 0.68 [0.47, 0.97] | -0.19 [-0.46, 0.08] | 0.17 | -0.32 [-0.62, -0.02] | 0.04 |
| Mixed | 23 | 0.87 [0.81, 0.93] | 0.03 [-0.18, 0.25] | 0.76 | -0.08 [-0.34, 0.18] | 0.54 |
| Histological type |  |  |  |  |  |  |
| SCC | 7 | 0.74 [0.52, 1.06] |  |  |  |  |
| Non-SCC | 9 | 0.74 [0.57, 0.96] | -0.02 [-0.34, 0.30] | 0.92 | 0.11 [-0.25, 0.47] | 0.57 |
| Mixed | 24 | 0.87 [0.81, 0.94] | 0.15 [-0.15, 0.44] | 0.36 | 0.01 [-0.32, 0.35] | 0.93 |
| PD-L1 status |  |  |  |  |  |  |
| ≥1% | 11 | 0.88 [0.76, 1.01] |  |  |  |  |
| <1% or NR | 29 | 0.81 [0.73, 0.90] | -0.08 [-0.26, 0.10] | 0.38 | -0.08 [-0.36, 0.19] | 0.56 |
| Treatment mode |  |  |  |  |  |  |
| ICI monotherapy | 20 | 0.85 [0.77, 0.92] |  |  |  |  |
| ICI combination | 19 | 0.79 [0.67, 0.94] | -0.07 [-0.24, 0.10] | 0.45 | -0.13 [-0.52, 0.25] | 0.50 |
| Mixed | 1 | 0.91 [0.68, 1.22] | 0.06 [-0.39, 0.51] | 0.79 | -0.29 [-0.89, 0.31] | 0.34 |
| ICI drug |  |  |  |  |  |  |
| Anti-PD-1 | 22 | 0.78 [0.67, 0.90] |  |  |  |  |
| Anti-PD-L1 | 13 | 0.85 [0.78, 0.94] | 0.09 [-0.08, 0.26] | 0.30 | 0.02 [-0.20, 0.24] | 0.85 |
| Others | 5 | 0.95 [0.82, 1.08] | 0.21 [-0.00, 0.43] | 0.06 | 0.37 [0.02, 0.72] | 0.04 |
| Treatment line |  |  |  |  |  |  |
| 1 line | 30 | 0.83 [0.75, 0.93] |  |  |  |  |
| ≥2 line | 10 | 0.81 [0.72, 0.91] | -0.02 [-0.20, 0.16] | 0.84 | -0.03 [-0.31, 0.25] | 0.85 |
| **PFS (female)** |  |  |  |  |  |  |
| Sample size |  |  |  |  |  |  |
| <100 | 11 | 0.75 [0.63, 0.91] |  |  |  |  |
| ≥100 | 19 | 0.75 [0.63, 0.88] | -0.00 [-0.29, 0.29) | 1.00 | -0.06 [-0.35, 0.23] | 0.68 |
| Risk bias |  |  |  |  |  |  |
| High | 18 | 0.80 [0.69, 0.93] |  |  |  |  |
| Low or unclear | 12 | 0.66 [0.54, 0.81] | -0.19 [-0.45, 0.06] | 0.14 | -0.07 [-0.41, 0.26] | 0.67 |
| Race |  |  |  |  |  |  |
| Asian | 12 | 0.69 [0.59, 0.81] |  |  |  |  |
| Non-Asian | 6 | 0.76 [0.53, 1.08] | 0.11 [-0.24, 0.45] | 0.54 | -0.17 [-0.52, 0.19] | 0.36 |
| Mixed | 12 | 0.79 [0.64, 0.96] | 0.14 [-0.16, 0.44] | 0.37 | -0.22 [-0.56, 0.12] | 0.20 |
| Histological type |  |  |  |  |  |  |
| SCC | 7 | 0.63 [0.50, 0.79] |  |  |  |  |
| Non-SCC | 10 | 0.66 [0.54, 0.80] | 0.04 [-0.31, 0.39] | 0.82 | 0.03 [-0.31, 0.38] | 0.84 |
| Mixed | 13 | 0.88 [0.74, 1.05] | 0.33 [-0.01, 0.68] | 0.06 | -0.03 [-0.48, 0.43] | 0.91 |
| PD-L1 status |  |  |  |  |  |  |
| ≥1% | 6 | 1.10 [0.84, 1.43] |  |  |  |  |
| < 1% or NR | 24 | 0.67 [0.60, 0.75] | -0.50 [-0.74, -0.25] | <0.001 | -0.49 [-0.93, -0.05] | 0.03 |
| Treatment mode |  |  |  |  |  |  |
| ICI monotherapy | 10 | 0.99 [0.83, 1.19] |  |  |  |  |
| ICI combination | 19 | 0.63 [0.56, 0.70] | -0.47 [-0.67, -0.27] | <0.001 | -0.13 [-0.80, 0.54] | 0.71 |
| Mixed | 1 | 0.76 [0.55, 1.05] | -0.28 [-0.73, 0.18] | 0.23 | -0.13 [-1.16, 0.89] | 0.80 |
| ICI drug |  |  |  |  |  |  |
| Anti-PD-1 | 20 | 0.74 [0.63, 0.87] |  |  |  |  |
| Anti-PD-L1 | 8 | 0.75 [0.57, 1.00] | 0.01 [-0.28, 0.30] | 0.93 | 0.14 [-0.19, 0.47] | 0.40 |
| Others | 2 | 0.79 [0.63, 0.98] | 0.06 [-0.41, 0.53] | 0.81 | 0.41 [-0.37, 1.19] | 0.30 |
| Treatment line |  |  |  |  |  |  |
| 1 line | 25 | 0.69 [0.61, 0.78] |  |  |  |  |
| ≥2 line | 5 | 1.04 [0.77, 1.40] | 0.41 [0.14, 0.69] | 0.004 | 0.29 [-0.15, 0.73] | 0.20 |

Abbreviations: RCTs, randomized controlled trials; OS, overall survival; PFS,progression-free survival; HR, hazard ratio; CI, confidence interval; NR, not reported; SCC, squamous cell carcinoma; ICI, immune checkpoint inhibitor.

**S4 Table** Previous meta-analyses assessing efficacy of ICIs by sex in NSCLC

| First author  /Published year | Total trials included (advanced NSCLC) | Treatment | Reported HR (95% CI) for OS | | | Conclusion |
| --- | --- | --- | --- | --- | --- | --- |
|  |  |  | males | females | P interaction |  |
| Wu/2018[5] | 11(4) | ICIs alone vs. CT | 0.62(0.53-0.71) | 0.74(0.65-0.84) | 0.07 | Significant sex-related efficacy difference was observed in melanoma but NSCLC patients. |
| Conforti/2018[6] | 20(6) | ICIs vs. CT or ICIs | 0.72(0.65-0.79) | 0.86(0.79-0.93) | 0.002 | ICIs can improve OS for advanced cancers, but the magnitude of benefit is sex-dependent. |
| Conforti/2019[7] | 8(7) | ICIs vs. CT | 0.76(0.66-0.87) | 0.48(0.35-0.67) | < 0.05 | Women with advanced lung cancer derived a larger benefit from CT plus ICIs as compared with men |
| Wang/2019[8] | 15(15) | ICIs vs. CT | 0.76(0.71-0.82) | 0.73(0.58-0.91) | NR | Male patients seemed to obtain more benefits from ICIs than female patients . |
| Wallis/2019[9] | 23(11) | ICIs vs. non-ICIs | 0.75(0.69-0.81) | 0.77(0.67-0.88) | 0.60 | No association of sex with the efficacy of ICIs was observed in the treatment of advanced cancers. |
| Yang/2020[10] | 37(14) | ICIs vs. CT | 0.75(0.71-0.81) | 0.79(0.72-0.88) | 0.25 | This study found no association of sex with cancer immunotherapy OS benefit. |
| Conforti/2021[11] | 4(4) | ICIs alone vs. CT | 0.59(0.50-0.69) | 0.84(0.64-1.10) | 0.04 | ICIs monotherapy was highly effective in men but not in women, even in case of high PD-L1 levels. |
| Xue/2021[12] | 21(21) | ICIs vs. CT | 0.73(0.67-0.79) | 0.73(0.61-0.85) | 0.97 | The interaction between ICIs efficacy and different sexes was equally evident. |
| Lai/2021[13] | 39(16) | ICIs vs. CT | 0.75(0.71-0.80) | 0.77(0.70-0.85) | 0.91 | No sex differences in the response to ICIs were found. |
| Madala/2022[14] | 16(16) | ICIs vs. CT | 0.74(0.66-0.83) | 0.72(0.63-0.82) | 0.71 | ICIs offer the same OS benefit in NSCLC regardless of gender. |
| Liang/2022[15] | 16(16) | ICIs vs. non-ICIs | 0.76(0.71-0.81) | 0.74(0.63-0.87) | 0.82 | Females seemed to benefit more from ICIs+CT than males, and males seemed to benefit more from ICIs alone than females. |
| Takada/2022[16] | 9(9) | ICIs combination vs. non-ICIs | 0.80(0.72-0.87) | 0.69(0.54-0.89) | NR | In patients receiving ICIs combination as a frst-line therapy, a better OS was observed in females than in males. |
| Lai/2024[17] | 108(34) | ICIs vs. non-ICIs | 1 line: 0.78(0.74-0.81)  2 line: 0.76(0.72-0.81) | 1 line: 0.82(0.77-0.87)  2 line: 0.89(0.80-0.99) | 1 line: 0.37  2 line: 0.004 | Sex should be taken into account when deciding to offer ICIs to patients who have already been heavily treated. |
| Zhong/2024[18] | 103(30) | ICIs vs. non-ICIs | 0.77(0.74-0.79) | 0.81(0.78-0.85) | 0.02 | ICI therapy has a positive impact on survival in cancers, and males benefit more than females. |
| Suazo-Zepeda/2026 [19] | 55(55) | ICIs vs. CT | 0.75(0.72-0.78) | 0.81(0.76-0.86) | 0.17 | ICIs confer similar OS benefits in men and women, but women show shorter PFS, particularly in PD-L1-positive and monotherapy settings. |

Abbreviations: ICIs, immune checkpoint inhibitors; CT, chemotherapy; NSCLC, non-small cell lung cancer; OS, overall survival; PFS, progression-free survival; HR, hazard ratios; CI, confidence intervals; NR, not reported.

**S1 Fig** Forest plot of overall survival for subgroup of Asian.

**S2 Fig** Forest plot of overall survival for subgroup of non-Asian.

**S3 Fig** Forest plot of overall survival for subgroup of SCC. SCC, squamous cell carcinoma.

**S4 Fig** Forest plot of overall survival for subgroup of non-SCC. SCC, squamous cell carcinoma.

**S5 Fig** Forest plot of overall survival for subgroup of PD-L1 ≥1%.

**S6 Fig** Forest plot of overall survival for subgroup of PD-L1 ≥50%.

**S7 Fig** Forest plot of overall survival for subgroup of 1 line.

**S8 Fig** Forest plot of overall survival for subgroup of ≥2 line.

**S9 Fig** Forest plot of overall survival for subgroup of ICI monotherapy. ICI, immune checkpoint inhibitor.

**S10 Fig** Forest plot of overall survival for subgroup of anti-PD-1 monotherapy.

**S11 Fig** Forest plot of overall survival for subgroup of anti-PD-L1 monotherapy.

**S12 Fig** Forest plot of overall survival for subgroup of ICI combination. ICI, immune checkpoint inhibitor.

**S13 Fig** Forest plot of overall survival for subgroup of ICI plus chemotherapy. ICI, immune checkpoint inhibitor.

**S14 Fig** Forest plot of overall survival for subgroup of dual ICIs. ICI, immune checkpoint inhibitor.

**S15 Fig** Forest plot of progression-free survival for subgroup of Asian.

**S16 Fig** Forest plot of progression-free survival for subgroup of non-Asian.

**S17 Fig** Forest plot of progression-free survival for subgroup of SCC. SCC, squamous cell carcinoma.

**S18 Fig** Forest plot of progression-free survival for subgroup of non-SCC. SCC, squamous cell carcinoma.

**S19 Fig** Forest plot of progression-free survival for subgroup of PD-L1 ≥1%.

**S20 Fig** Forest plot of progression-free survival for subgroup of PD-L1 ≥50%.

**S21 Fig** Forest plot of progression-free survival for subgroup of 1 line.

**S22 Fig** Forest plot of progression-free survival for subgroup of ≥2 line.

**S23 Fig** Forest plot of progression-free survival for subgroup of ICI monotherapy. ICI, immune checkpoint inhibitor.

**S24 Fig** Forest plot of progression-free survival for subgroup of anti-PD-1 monotherapy.

**S25 Fig** Forest plot of progression-free survival for subgroup of anti-PD-L1 monotherapy.

**S26 Fig** Forest plot of progression-free survival for subgroup of ICI combination. ICI, immune checkpoint inhibitor.

**S27 Fig** Forest plot of progression-free survival for subgroup of ICI plus chemotherapy. ICI, immune checkpoint inhibitor.

**S28 Fig** Sensitivity analysis sequentially omitting one trial at a time for overall survival in males.

**S29 Fig** Sensitivity analysis sequentially omitting one trial at a time for overall survival in females.

**S30 Fig** Sensitivity analysis sequentially omitting one trial at a time for progression-free survival in males.

**S31 Fig** Sensitivity analysis sequentially omitting one trial at a time for progression-free survival in females.

**S32 Fig** Sensitivity analysis excluding trials with low representation of female patients for overall survival.

**S33 Fig** Sensitivity analysis excluding trials with low representation of female patients for progression-free survival.

**S34 Fig** Assessment of risk of bias. a: Methodological quality graph: authors’ judgment about each methodological quality item presented as percentages across all included studies; b: Methodological quality summary: authors’ judgment about each methodological quality item for each included study, “+” low risk of bias; “?” unclear risk of bias; “-” high risk of bias. RCTs, randomized controlled trials.

**S35 Fig** Funnel plots of publication bias.
